# Mapping key neuropeptides involved in the melanocortin system in Atlantic salmon (*Salmo salar*) brain

**DOI:** 10.1002/cne.25415

**Published:** 2022-10-10

**Authors:** Sissel Norland, Mariann Eilertsen, Ivar Rønnestad, Jon Vidar Helvik, Ana S. Gomes

**Affiliations:** ^1^ Department of Biological Sciences University of Bergen Bergen Norway

**Keywords:** Agrp, appetite, Atlantic salmon, Cart, hypothalamus, Npy, Pomc

## Abstract

The melanocortin system is a key regulator of appetite and food intake in vertebrates. This system includes the neuropeptides neuropeptide y (NPY), agouti‐related peptide (AGRP), cocaine‐ and amphetamine‐regulated transcript (CART), and pro‐opiomelanocortin (POMC). An important center for appetite control in mammals is the hypothalamic arcuate nucleus, with neurons that coexpress either the orexigenic NPY/AGRP or the anorexigenic CART/POMC neuropeptides. In ray‐finned fishes, such a center is less characterized. The Atlantic salmon (*Salmo salar*) has multiple genes of these neuropeptides due to whole‐genome duplication events. To better understand the potential involvement of the melanocortin system in appetite and food intake control, we have mapped the mRNA expression of *npy*, *agrp*, *cart*, and *pomc* in the brain of Atlantic salmon parr using in situ hybridization. After identifying hypothalamic mRNA expression, we investigated the possible intracellular coexpression of *npy/agrp* and *cart/pomc* in the tuberal hypothalamus by fluorescent in situ hybridization. The results showed that the neuropeptides were widely distributed, especially in sensory and neuroendocrine brain regions. In the hypothalamic lateral tuberal nucleus, the putative homolog to the mammalian arcuate nucleus, *npya*, *agrp1*, *cart2b*, and *pomca* were predominantly localized in distinct neurons; however, some neurons coexpressed *cart2b/pomca*. This is the first demonstration of coexpression of *cart2b/pomca* in the tuberal hypothalamus of a teleost. Collectively, our data suggest that the lateral tuberal nucleus is the center for appetite control in salmon, similar to that of mammals. Extrahypothalamic brain regions might also be involved in regulating food intake, including the olfactory bulb, telencephalon, midbrain, and hindbrain.

## Graphical abstract

*npy*, *agrp*, *cart*, and *pomc* were expressed in feeding‐related regions, including hypothalamus of Atlantic salmon (*Salmo salar*). In the hypothalamic lateral tuberal nucleus, the putative homolog to the mammalian arcuate nucleus, *npya*, *agrp1*, *cart2b*, and *pomca* were predominantly localized in distinct neurons; however, some neurons coexpressed *cart2b/pomca*.

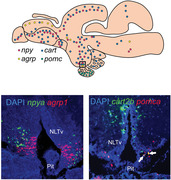

**Abbreviations**
CC: cerebellar corpus
Ce: nucleus centralis lobi inferioris hypothalami
Cho: optic chiasm
Cp: commissura posterior
D: dorsal telencephalon
Dc: central zone of dorsal telencephalon
Dd: dorsal zone of dorsal telencephalon
Dl‐d: dorsal part of lateral zone of dorsal telencephalon
Dl‐v: ventral part of lateral zone of dorsal telencephalon
Dm: medial zone of dorsal telencephalon (dorsal pallium)
DTN: dorsal tegmental nucleus
ent: nucleus entopeduncularis
EW: Edinger–Westphal nucleus
FLM: medial longitudinal fasciculus
fMth: fiber of Mauthner cell
Ggl: stratum ganglionare (cerebellum)
Gran: stratum granulare (cerebellum)
Hab: habenula
inf: infundibulum
Lcoer: locus coeruleus
Lih: inferior hypothalamic lobe
Mc: layer of mitral cells (bulbi olfactori)
Mcba: tractus mesencephalo‐cerebellaris anterior
Mfb: medial forebrain bundle
mol: stratum moleculare (cerebellum)
NAT: nucleus anterior tuberis
NDILm: medial part of the diffuse nucleus of inferior lobe
nIII: nucleus oculomotorius
NLT: nucleus lateralis tuberis
NLTa: anterior nucleus lateralis tuberis
NLTp: posterior nucleus lateralis tuberis
NLTv: ventral nucleus lateralis tuberis
NLV: nucleus lateralis valvulae
NMFL: nucleus medial longitudinal fasciculus
NMH: nucleus magnocellularis hypothalami
NPP: nucleus posterioris periventricularis
NPT: nucleus posterior tuberis
Nrl: nucleus recessi lateralis
nV: nervi trigemini
nVm: nucleus motorius nervi trigemini
OT: Optic tectum
Pit: pituitary
Ppa: preoptic area—anterior parvocellular preoptic nucleus
Ppp: preoptic area—posterior parvocellular preoptic nucleus
Psp: nucleus pretectal superficialis magnocellularis
Pt: posterior tuberculum
PTN: nucleus posterior tuberis
PVO: paraventricular organ
Retm: formatio reticularis pars medialis
Rets: formatio reticularis pars superior
RF: reticular formation
rl: recessi lateralis
Rpo: recessus preopticus
SAC: stratum album centrale (tecti mesencephali)
SGC: stratum griseum centrale (tecti mesencephali)
SM: stratum marginale (tecti mesencephali)
SO: stratum opticum (tecti mesencephali)
SOC: supraoptic/suprachiasmatic nucleus
SPV: stratum periventriculare (tecti mesencephali)
Stgr: stratum granulare (bulbi olfactori)
SV: saccus vasculosus
Tbc: tractus tecto‐bulbaris cruciatus
Thd: dorsal thalamus
Thv: ventral thalamus
TL: Torus longitudinalis
Tlat: torus lateralis
TLw: white matter region of torus longitudinalis
Tod: tractus opticus dorsalis
Toll: tractus olfactorius lateralis
TS: Torus semicircularis
Valv: Valvula cerebelli
Vd: dorsal nucleus of ventral telencephalon
Ve4: fourth ventricle (rhombencephali)
vHab: ventral habenula
Vl: lateral nucleus of ventral telencephalon
Vv: ventral nucleus of ventral telencephalon

## 1 INTRODUCTION

In fish, like in other vertebrates, appetite and food intake are controlled by endocrine signals and neuropeptides released in neural pathways in the brain (Comesaña et al., 2018; Rønnestad et al., 2017; Soengas et al., 2018; Volkoff, 2016). The neuronal network receives continuous feedback from peripheral tissues, especially the gastrointestinal tract, liver, and pancreas, where nutrients and endocrine and neuronal signals interact to regulate food intake and energy balance (Rønnestad et al., 2017). Food intake is also controlled by sensory and hedonic inputs, such as liking and wanting, that drive hunger and satiety. These inputs originate from a motivation/reward center with dopaminergic neurons (Palmiter, 2007; Soengas et al., 2018); the neurons assist in the underlying mechanism of food intake through conditioning, chemosensory stimulation from the smell of food (Rossi & Stuber, 2018), or nutrient sensing in the brain (Comesaña et al., 2018).

The melanocortin system is a key player in neuronal appetite control. In mammals, it is well characterized and comprises two major neuronal circuits in the arcuate nucleus of the hypothalamus (Elias et al., 1998; Hahn et al., 1998; Schwartz et al., 2000). These neurons are known to either stimulate (orexigenic) or inhibit (anorexigenic) appetite. Both orexigenic and anorexigenic neurons are competitively interacting with the melanocortin receptors (Nuzzaci et al., 2015). Coexpression of neuropeptide y (NPY) and agouti‐regulated peptide (AGRP) increases orexigenic activity resulting in an anabolic response. In contrast, neurons coexpressing cocaine‐ and amphetamine‐regulated transcript (CART) and pro‐opiomelanocortin (POMC) act as anorexigenic, providing a catabolic response.

The key neuropeptides involved in the melanocortin system described in mammals have also been identified in teleosts (Delgado et al., 2017; Rønnestad et al., 2017; Soengas et al., 2018; Volkoff, 2016; Volkoff et al., 2005). However, teleosts typically possess multiple paralogs of these genes compared to mammals due to the additional teleost‐specific whole‐genome duplication, and some teleost families, like salmonids, have additional copies because of the salmonid‐specific fourth round of whole‐genome duplication event (Allendorf & Thorgaard, 1984; Lien et al., 2016). For most of these paralogs, their effects on appetite and food intake control remain unclear.

NPY is a potent and abundant orexigenic factor in the brain and plays a key role in energy homeostasis and food intake in mammals (Loh et al., 2015), as well as in several teleosts (Volkoff, 2016). Earlier studies on Atlantic salmon, *Salmo salar*, supported the involvement of *npy* in food intake control (Murashita et al., 2009; Valen et al., 2011). Recently, three *npy* paralogs have been identified in the Atlantic salmon, named *npya1*, *npya2*, and *npyb* (Tolås et al., 2021). Tolås et al. (2021) showed that neither of the *npy* paralogs was significantly affected by feeding status in the hypothalamus, albeit a trend of increased *npya2* mRNA expression following 4 days of fasting was observed. AGRP is also a key player in the orexigenic melanocortic pathway (Morton & Schwartz, 2001). In Atlantic salmon, Murashita et al. (2009) identified two Agouti‐like sequences, named *agrp1* and *agrp2* (also named *asip2b*, see Braasch and Postlethwait (2011) and NCBI GenBank^1^). The orexigenic effect of Atlantic salmon *agrp1* seems to be in line with those reported in mammals (Kalananthan, Murashita, et al., 2020). However, *agrp2* seems to not be directly involved in appetite control in Atlantic salmon (Kalananthan, Lai, et al., 2020) but may play other functional roles, as demonstrated in the zebrafish, *Danio rerio* (Shainer et al., 2017, 2019; Zhang et al., 2010).

CART is a neuropeptide involved in several processes in the brain, including appetite control. Mammals have one *cart* gene that plays an anorexigenic role (Akash et al., 2014). However, there are 10 *cart* paralogues in Atlantic salmon with varying and differential expressions in different brain regions, and their full physiological function(s) are not fully established (Kalananthan et al., 2021). POMC is a precursor peptide that is post‐translationally cleaved into several peptides with a wide range of functions, including α‐ and β‐melanocyte‐stimulating hormones, adrenocorticotropic hormone, and β‐endorphin (Takahashi & Mizusawa, 2013). Three *pomc* paralogs (*pomca1*, *pomca2*, and *pomcb*) have been previously identified in Atlantic salmon (Murashita et al., 2011; Valen et al., 2011) and are primarily expressed in the pituitary and hypothalamus (Kalananthan, Lai, et al., 2020).

The topology of central neuropeptides of the melanocortin system has been mapped in the whole brain or in specific brain regions in several teleost species, including catfish *Clarias batrachus* (Gaikwad et al., 2004; Singru et al., 2008; Subhedar et al., 2011), Indian major carp *Cirrhinus cirrhosus* (Saha et al., 2015), goldfish *Carassius auratus* (Cerdá‐Reverter & Peter, 2003; Cerdá‐Reverter, Schiöth, et al., 2003; Kojima et al., 2010; Matsuda et al., 2009), zebrafish (Akash et al., 2014; Forlano & Cone, 2007; Jeong et al., 2018; Kaniganti et al., 2021; Koch et al., 2019; Mukherjee et al., 2012; Shainer et al., 2017, 2019), sea bass *Dicentrarchus labrax* (Agulleiro et al., 2014; Cerdá‐Reverter et al., 2000), Atlantic cod *Gadus morhua* (Le et al., 2016), and the African cichlid fish *Astatotilapia burtoni* (Porter et al., 2017). However, to our knowledge, coexpression of *npy/agrp* and *cart/pomc* in the hypothalamus has never been observed in a teleost species. In salmonids, *pomc* and *agrp* have been identified in the hypothalamus of rainbow trout *Oncorhynchus mykiss* (Otero‐Rodino et al., 2019). Npy expression has been documented in the brown trout *Salmo trutta fario* brain including the dorsal and ventral telencephalon, habenula, periventricular and tuberal hypothalamus, saccus vasculosus, tectum, tegmentum, and the rhombencephalon (Castro et al., 1999). In Atlantic salmon and Gambusia affinis brain, Npy expression was found in the ventral telencephalon, tectum, tegmentum, and rhombencephalon (Garcia‐Fernandez et al., 1992). However, the spatial distribution of these melanocortin neuropeptides has not been fully explored in the whole brain of salmonids. Atlantic salmon is an important aquaculture species and understanding the systems that control appetite and food intake is central to optimize their feeding regimes. Taking into consideration that appetite is controlled by neuronal circuits in the brain, mapping the various neuroendocrine cell clusters in the different brain regions is key to uncover the melanocortin system contribution.

In this study, we have described the mRNA expression of *npy*, *agrp*, *cart*, and *pomc* genes in the Atlantic salmon parr brain by in situ hybridization (ISH). Next, to identify potential key neural circuits involved in appetite control, we investigated the possible coexpression of putative anorexigenic and orexigenic neuropeptides in the Atlantic salmon tuberal hypothalamus.

## 2 MATERIALS AND METHODS

### 2.1 Ethical statement

The Atlantic salmon were obtained from the Industrial and Aquatic Laboratory (Bergen High Technology Center, Norway) which has all the necessary approvals for running trials on fish. Atlantic salmon were reared following the Norwegian Veterinary Authorities’ standard protocols. The fish did not undergo any treatment or handling except for euthanasia; thus, special approval from the food authorities and ethics committee was deemed unnecessary according to Norwegian National legislation via the Norwegian Animal Welfare Act (LOV‐2015‐06‐09‐16‐65) and Regulations on the Use of Animals in Experiments (FOR‐2017‐04‐05‐ 451), as required in the European Union (Directive 2010/63/EU) for animal experiments. All fish used were euthanized with an overdose of MS‐222 (MS‐222™; MSD Animal Health, the Netherlands) on site, before further handling.

### 2.2 Sampling

For RNA extraction and cloning, the brain and pituitary were dissected from one Atlantic salmon (weight = 900 g, standard length = 38.5 cm), stored in RNAlater (Invitrogen, Carlsbad, USA) at 4°C overnight, and then transferred to −80°C. For ISH, 18 Atlantic salmon parr (weight = 33.7 ± 3.5 g, standard length = 14.1 ± 0.5 cm) were killed with an overdose of 200 mg/L MS‐222. An incision was made mid‐ventral to expose the heart for whole‐animal perfusion fixation with 4% paraformaldehyde (PF) in phosphate‐buffered saline (PBS) pH 7.4 (4% PF). Thereafter, brains were carefully dissected out of the skull and post‐fixed in 4% PF for 48 h, rinsed in 1× PBS, and immersed in 25% sucrose/25% OCT (CellPath, UK) for 24 h as described in Eilertsen et al. (2021). The brains were embedded in 100% OCT and coronal parallel cryosectioned across the entire extent of the brain at 10 μm using Leica CM 3050s cryostat (Leica Biosystems, Germany) and collected on SuperFrost Ultra Plus glasses (Menzel Glaser, Germany). Sections were dried at 65°C for 30 min and stored at −20°C until analyzed by ISH.

### 2.3 RNA extraction and cDNA synthesis

Total RNA was isolated from both Atlantic salmon brain and pituitary using TRI reagent (MilliporeSigma, St. Louis, USA) following the manufacturer's instructions, and further treated with TURBO DNA‐free (ThermoFisher Scientific, Indianapolis, USA). First‐strand cDNA was synthesized from 1.5 μg of DNase‐treated total RNA using oligo(dT)20 primer from SuperScript III First‐Strand Synthesis system for RT‐PCR kit (ThermoFisher Scientific).

### 2.4 Molecular cloning

Primer design was done in ApE‐A plasmid editor (http://biologylabs.utah.edu/jorgensen/wayned/ape/, RRID: SCR_014266). Primers and product sizes are listed in Table 1. Atlantic salmon *npya*, *npyb*, *agrp1*, *agrp2*, *pomca*, *pomcb*, *cart1b*, and *cart2b* amplification was performed with Advantage 2 PCR kit (Clontech, Mountain View, CA, USA) using Advantage SA buffer. PCR amplification was performed using a BIO‐RAD C1000 Touch Thermal Cycler (Bio‐Rad, Germany) with an initial step of 95°C for 3 min, and 34 cycles of 30 s denaturation at 95°C, 30 s annealing at 58–60°C, and 1 min extension at 68°C ending with a final extension at 68°C for 10 min.

**TABLE 1:** List of primers used for molecular cloning of genes involved in the melanocortin system in Atlantic salmon

Target gene	Accession number	Probe length (bp)	Primer sequence (5′−3′)
*npya*	*npya1* (**NM_001146681**)	359	F: GCCTGAGGACAACTTCTATC
	*npya2* (XM_014178359)		R: GACACTATTACCACAACGACG
*npyb*	*npyb* (**XM_045697117** and XM_014184208)	423	F: GCGAGCACAGAACAGTCATTC
			R: GTGGTGTTGTGACAAACAGGC
*agrp1*	*agrp1* (**NM_001146677**)	612	F: GAAGCGCTTTGTTGCATCAGC
			R: GTACACCCAACGTAACATCCATC
	T3 and T7 primers for agrp1 probe		T3: CATTAACCCTCACTAAAGGGAAGAAGCGCTTTGTTGCATCAGC
			T7: TAATACGACTCACTATAGGGCTATAGGCCCCACCTCATGGA
*agrp2/asip2b*	*agrp2* (**NM_001146678**)	479	F: GAGCGAGAACATTCTGAGCTG
			R: GTCTAGGTCTTCTTGGGGCAG
*cart1a*	*cart1a* (**XM_014149393**)	482	F: CGTATAAAACCTTGGTCCAGG
			R: CATACAACATTGAGTCATCCCG
*cart1b*	*cart1b1* (**XM_014150559**)	618	F: CTGTATCTCCATCCCTTCTG
	*cart1b2* (XM_014151634)		R: GACAACAAACCCTCCATTAC
*cart2a*	*cart2a* (**ENSSSAG00000015472**)	894	F: ATGGAGAGCTCTAAACTGTGGA
			R: CACAAGCACTTCAACAGAAAGAAG
*cart2b*	*cart2b1* (NM_001146680)	567	F: CGGGACCTTTTGGAGACGAAA
	*cart2b2* (**XM_014183838**)		R: TGGGGTTTGGACAATCTCTCAG
*cart3a*	*cart3a1* (XM_014177116)	585	F: GAACTGCAAATTAGAGAGGGAG
	*cart3a2* (**NM_001141227**)		R: TCAAGACAGTCATACATGCAG
*cart3b*	*cart3b* (**XM_014127320**)	389	F: CATTGGGAAGCTCTGTGAC
			R: GCTGTAAATGCTTTCTGGG
*cart4*	*cart4* (**XM_014141614)**	811	F: GCCTACAGCTTGTGTCAACC
			R: GACGTACTGGGAAAGTGTTCAT
*pomca*	*pomca1 (*NM_001198575)	689	F: GTTCTGACCTCACCGCCAAA
	*pomca2 (* **NM_001198576** )		R: GAGCTAACTGGCTCTAAGTCCT
*pomcb*	*pomcb* (**NM_001128604**)	624	F: AGGTAGTCCCCAGAACCCTC
			R: CAGTACGGTTCTCCGCTTCTT

*Note*: Accession numbers from GenBank or Ensembl are provided for the different target genes. In bold are the genes on which the probe synthesis was based.

*cart1a*, *cart3a*, *cart3b*, and *cart4* were amplified with Q5 High Fidelity 2X polymerase (New England Biolabs, Ipswich, MA, USA) using the following conditions: 98°C for 30 s; 34 cycles of 98°C for 10 s, 60°C for 20 s, 72°C for 30 s; and a final step at 72°C for 2 min.

PCR amplicons were purified from agarose gel using the MinElute Gel Extraction kit (Qiagen, Hilden, Germany) according to the manufacturer's protocol before being cloned into a StrataClone PCR cloning vector (Agilent Technologies, CA, USA). The products were sequenced at the University of Bergen Sequencing Facility using BigDye protocol (BigDye™ Terminator v3.1, ThermoFisher Scientific). Sequence identity was confirmed in Vector NTI software to ensure 100% sequence identity with the public available sequences.

### 2.5 Riboprobe

The cloned inserts were used to synthesize antisense and sense (control) digoxigenin (DIG)‐labeled (MilliporeSigma) and fluorescein (FITC)‐labeled (Roche Diagnostics, Germany) riboprobes. Synthesis was carried out as described in Thisse and Thisse (2008) using PCR products with added T3 (5′‐CATTAACCCTCACTAAAGGGAA‐3′) and T7 (5′‐TAATACGACTCACTATAGGG‐3′) on the forward and reverse cloning primers for sense and anti‐sense riboprobes, respectively (Table 1). The one exception was the T7 primer for *agrp1* (5′‐TAATACGACTCACTATAGGGCTATAGGCCCCACCTCATGGA‐3′). The RNA probes were precipitated using 4 M LiCl, 1 μg/μl tRNA (Roche Diagnostics), and 100% EtOH. When two paralogue genes shared a high sequence identity of the targeted region (> 92% identity level), one template was used in the riboprobe synthesis as the probe will label both genes. Note that for the paralogs of *pomca*, the overall sequence similarity of the target region was 81%; however, large fragments of the probe will hybridize to both genes (100% identity between 240–543 bp and 613–928 bp).

### 2.6 In situ hybridization

ISH was carried out using a modified protocol from Sandbakken et al. (2012) and replacing 50% deionized formamide with 4 M urea. In summary, sections were dried at room temperature for 30 min, and then at 65°C for 30 min before being rehydrated using an ethanol series (95%–50%). Afterward, sections were permeabilized with 10 μg/ml proteinase K (MilliporeSigma), post‐fixed in 4% PF, and treated with 0.25% acetic anhydride (MilliporeSigma) in 0.1 M triethanolamine (MilliporeSigma), ending with dehydration using an ethanol series (50%−100%).

The hybridization was carried out with a DIG‐labeled RNA probe overnight at 65°C. After hybridization, sections were washed and treated with RNase A (0.02 mg/ml, MilliporeSigma) before being incubated with sheep polyclonal anti‐DIG antibody (anti‐digoxigenin‐alkaline phosphatase FAB‐fragment, 1:2000, cat. # 11093274910, Roche Diagnostics, RRID: AB_514497) in 1× blocking solution (MilliporeSigma) overnight at room temperature. The result was visualized using 4‐Nitro blue tetrazolium chloride and 5‐Bromo‐4‐chloro‐3‐indolyl‐phosphate system (NBT/BCIP Ready‐to‐use tablets, MilliporeSigma). Parallel sections were Nissl stained with cresyl fast violet (Chroma‐Gesellschaft, Germany). Sections were rehydrated in an ethanol series (96%–50%), dipped in staining solution (0.35% cresyl violet), differentiated in 70% ethanol, and dehydrated in 100% ethanol (2 × 5 min) ending with clearing in xylene. For all genes, sense probes were applied as a control for nonspecific staining.

### 2.7 Double labeling fluorescence ISH

To investigate the coexpression of *npy* and *agrp* or *cart* and *pomc* in the tuberal hypothalamus, fluorescence double labeling ISH was done as described in Eilertsen et al. (2018), and replacing 50% deionized formamide with 4 M urea. DIG‐labeled riboprobes were incubated with sheep polyclonal anti‐DIG antibody (anti‐digoxigenin‐alkaline phosphatase FAB‐fragment, 1:2000, cat. # 11093274910, Roche Diagnostics, RRID: AB_514497) and detected with either Fast Red tablet (Roche Diagnostics) dissolved in 0.1 M Tris‐HCl pH 8.2 and 0.1% Tween‐20 or with 100 mg/ml Fast Blue BB salt (MilliporeSigma) and 100 mg/ml naphthol AS‐MX phosphate (MilliporeSigma) in 0.1 M Tris‐HCl pH 8.2, 50 mM MgCl_2_, 0.1 M NaCl, and 0.1% Tween‐20 (MilliporeSigma). A 2% blocking solution (MilliporeSigma) in 2× saline‐sodium citrate buffer was used for blocking the sections, followed by the visualization of FITC‐labeled riboprobes using sheep polyclonal anti‐FITC (anti‐fluorescein conjugated with horseradish peroxidase, Fab fragments, cat. # 1142636910, Roche, RRID: AB_840257) and TSA™ Fluorescein (Akoya Biosciences Marlborough, USA) according to the manufacture's protocol. Sections were mounted with ProLong Glass antifade medium with NucBlue (Invitrogen).

### 2.8 Microscopy

Whole sections were scanned at 20×/0.8 objective with ZEISS Axio Scan.Z1 Slide scanner (Zeiss, Germany, RRID: SCR_020927) and ZEN software (Zeiss). The setting for NBT/BCIP was in TL brightfield (BF) using Hitachi HV‐F202SCL. Fluorescent sections were scanned with DAPI, AF488 (TSA staining for *npya* and *cart2b*), AF568 (FastRed for *pomca*), and AF647 (FastBlue for *agrp1*), and using the Hamamatsu Orca Flash imaging device.

Confocal images were acquired by a laser‐scanning confocal microscope (Olympus FV3000, Olympus, Japan, RRID: SCR_017015) with 10× and 60× silicon‐immersion oil objective (UPLSAPO 40XS, Olympus), using DAPI, AF488 (TSA staining for *npya* and *cart2b*), AF568 (FastRed for *pomca*), and AF647 (FastBlue for *agrp1*). Image stacks from each channel were imported into Fiji (https://fiji.sc/, RRID: SCR_002285; Schindelin et al., 2012) to create z‐projections based on maximum intensity.

Figures were prepared using Adobe Photoshop (version 22.1.1, Adobe Systems, San Jose, CA, RRID: SCR_014199). The background was removed, and brightness and contrast were adjusted if necessary. The rainbow trout, *O. mykiss*, was used for reference and nomenclature of the brain regions in this study (Folgueira et al., 2004a, 2004b; Meek & Nieuwenhuys, 2014).

## 3 RESULTS

To map the expression of the neuropeptides involved in the melanocortin system in Atlantic salmon parr brain, *npy*, *agrp*, *cart*, and *pomc* mRNA were examined across the entire rostrocaudal extent of the brain by ISH in coronal parallel cryosections. A summary of the results is shown in Table 2.

**TABLE 2:** Summary of the mRNA expression of *npy*, *agrp*, *cart*, and *pomc* in the Atlantic salmon brain

Gene	Telencephalic regions	Diencephalic regions	Pituitary	Midbrain	Hindbrain
*npya*	MC, Vd, Vl	Ppa, SOC, vHab, Thv, Thd, Pt, NAT, NLTa, NLTv, NMH, Lih NLTp/NPT		SPV, SGC, EW*	FLM*, nV*
*npyb*	Vd, Vl	Thd		FLM*	
*agrp1*		NLTv, NLTp/NPT			
*agrp2*	Dm, Dc, Dl‐v	Thv			nV*, RF*
*cart1a*				NFLM*, nIII*	
*cart1b*				NFLM*	
*cart2a*	Vv,	Tod, Thd, PTN		SPV, SAC, SO	
*cart2b*	Stgr, Vv, Vd, Vl, Dm, Dl, Dd	Ppp, NAT, NLTa, SPV, TS, Tlat, Ce, NLTv, NMH, Lih, PVO, PRN, NRL, NDILm, SV		Gran, lcoer, Rets	
*cart3a*	Vv, Ent	SOC, Thd, NMH/PVO		TLw, EW*	FLM*, lcoer*, nV*
*cart3b*		Ppp, Thd		tlat, TS	FLM*, nV*, rets*
*cart4*		ppp			
*pomca*		NLTa, NLTv, NLTp/NPT	pit		
*pomcb*			pit		

* Cells expressing this gene near the indicated brain region. See Section 3 more details.

### 3.1 neuropeptide y (npy)

#### 3.1.1 npya

*npya* was widely distributed throughout the brain (Figure 1). In the olfactory bulb, *npya* expression was found lateroventrally in the mitral cell layer (mc, Figure 1b(1–2)). A high density of *npya* is further seen in the lateral nucleus of the ventral telencephalon (Vl). Medial scattered neuronal clusters in the dorsal nucleus of ventral telencephalon (Vd, Figure 1c(1–2)), and scattered cells in the posterior dorsal telencephalon (Figure 1(d1)) also expressed *npya*. Cells expressing *npya* were identified in the preoptic region, including ventral to the *recessus preopticus* (rpo, Figure 1(d2)), and supraoptic/suprachiasmatic nucleus (SOC, Figure 1e(1–2)). *npya* was expressed in the ventrolateral habenula (vHab) and thalamic regions including the ventral (Thv) and dorsal thalamus (Thd), and the posterior tuberculum (Pt, Figure 1f(1–2) and Figure 1g(1–3)).

FIGURE 1
*npya* mRNA expression in Atlantic salmon parr brain. (a) Schematic representation of the brain indicating the position of each transverse section. (b1–l1) Nissl‐staining compared to schematic drawing illustrating *npya* expression by green dots. (b2–l2, g3–j3) Nissl‐staining and corresponding *npya* expression along with neuroanatomical structures. (b) *npya* expression in the mc of the olfactory bulb. (c) *npya* expression in the Vd, and Vl of the telencephalon. (d) *npya* expression in dorsal telencephalon and preoptic area—ppa. (e) *npya* expression in the SOC. (f) *npya* expression in the vHab and Thv. (g) *npya* expression in the SPV, Thv, Pt, NAT, and NLTv. (h) *npya* expression in the SPV, SGC, and NMH. (h) *npya* expression in the Thd and NMH. (j) *npya* expression in the dorsal tegmentum near EW. (k) *npya* expression near FLM. (l) *npya* expression near nV. Abbreviations; Cho, optic chiasm; D, dorsal telencephalon; EW, Edinger–Westphal nucleus; FLM, fasciculus longitudinalis medialis; Hab, habenula; Lih, lobus inferior hypothalami; mc, layer of mitral cells; NAT, nucleus anterior tuberis; NLT, nucleus lateralis tuberis; NLTv, ventral nucleus lateralis tuberis; NMH, nucleus magnocellularis hypothalami; nV, nervus trigemini; Ppa, preoptic area—anterior parvocellular preoptic nucleus; Pt, posterior tuberculum; Rpo, recessus preopticus; SGC, stratum griseum centrale; SM, stratum marginale; SOC, supraoptic/suprachiasmatic nucleus; SPV, stratum periventriculare of the optic tectum; stgr, stratum granulare (bulbi olfactory); Thd, dorsal thalamus; Thv, ventral thalamus; Ts, torus semicircularis; Valv, valvula cerebelli; Vd, dorsal nucleus of ventral telencephalon; vHab, ventral habenula; Vl, lateral nucleus of ventral telencephalon; Vv, ventral nucleus of ventral telencephalon. Scale bar (if no other indication) = 500 μm

**Figure:**
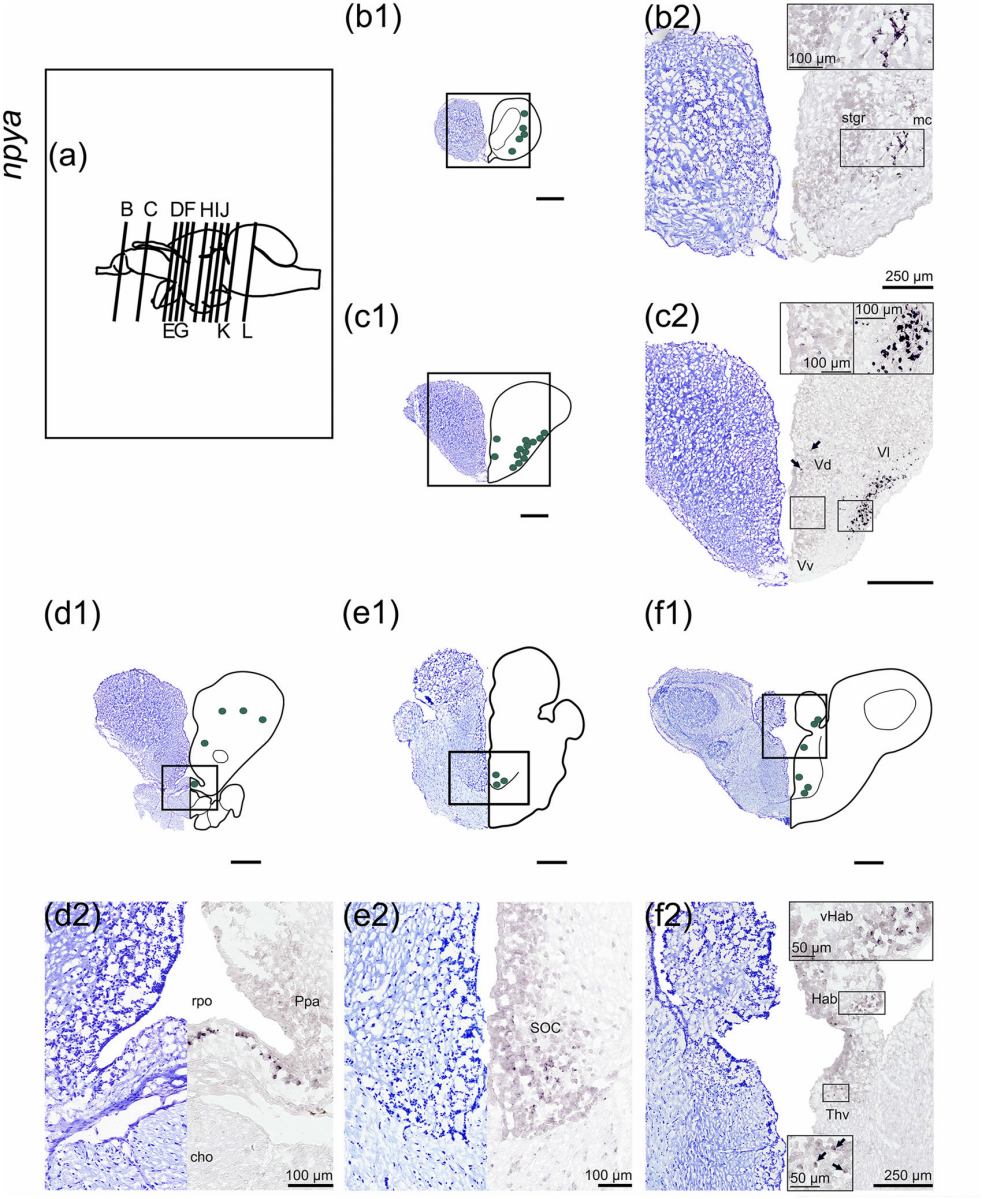

**Figure:**
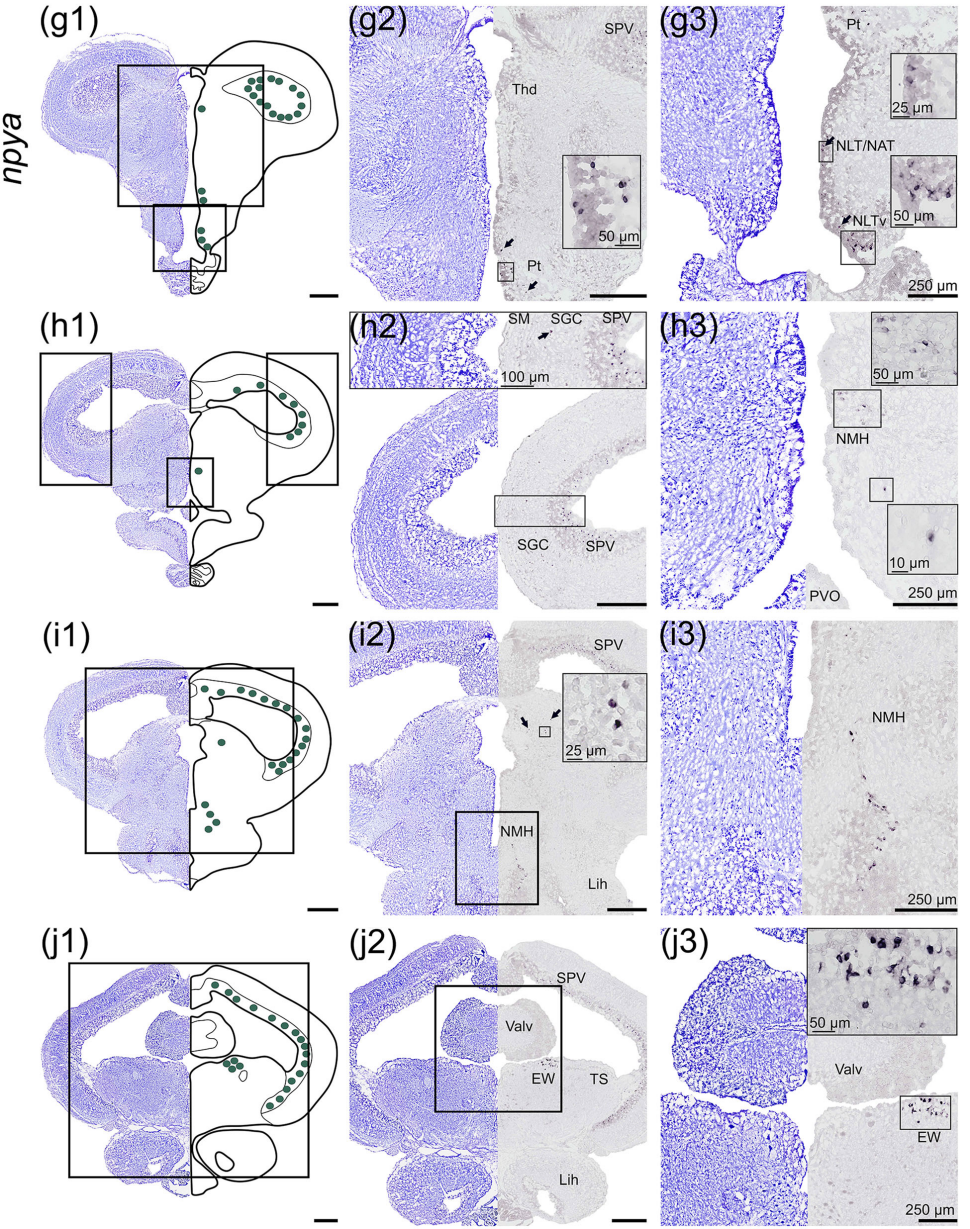

**Figure:**
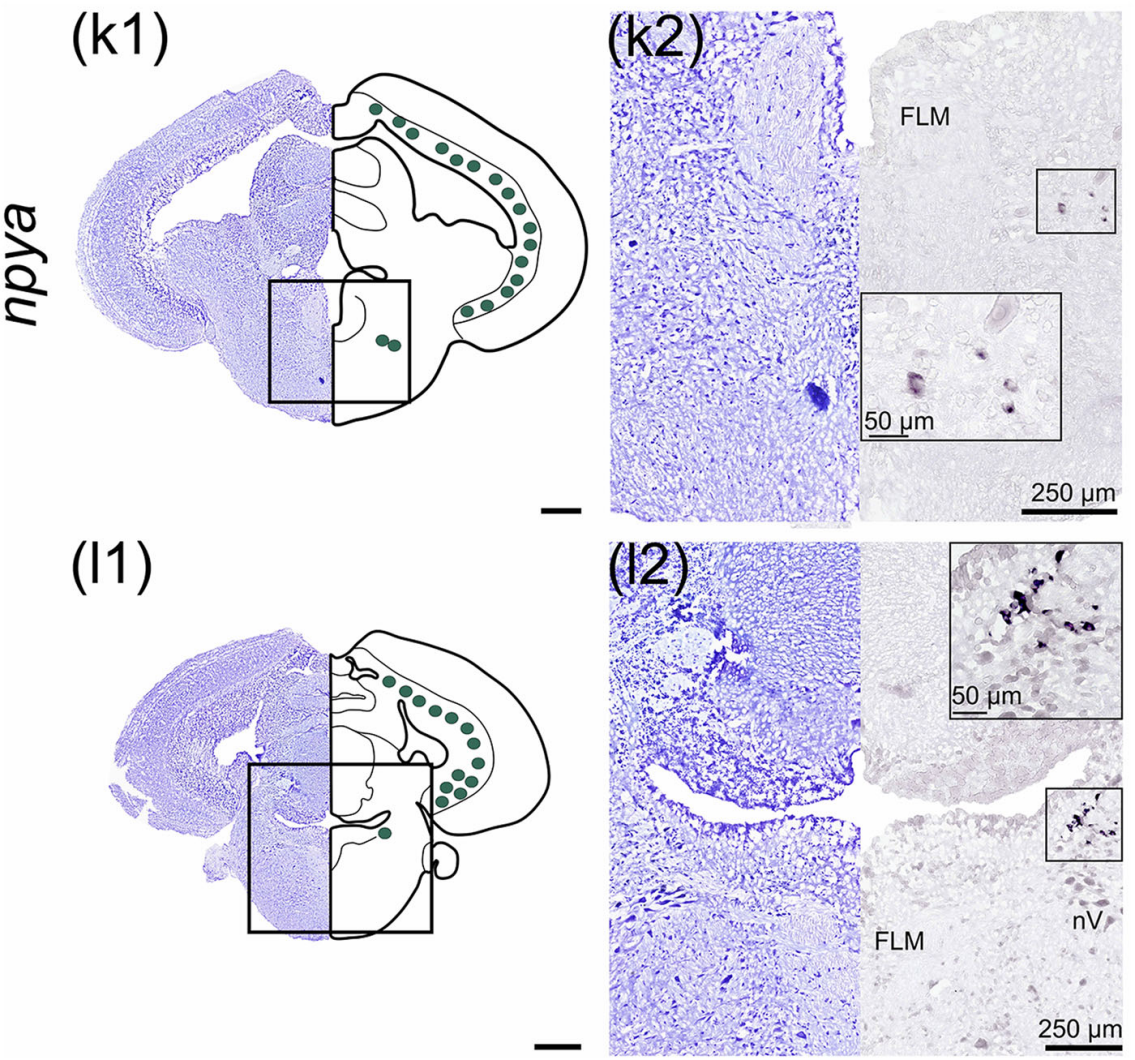

Hypothalamic *npya* expression was observed at the pituitary stalk in ventral nucleus lateralis tuberis (NLTv), nucleus anterior tuberis (NAT), and nucleus magnocellularis hypothalami (NMH, Figure 1g(1–3) (h3), and (i3)). In the optic tectum, *npya* was expressed in the periventricular layer (SPV) and in a few cells in the griseum layer (SGV, Figure 1(h2)). Positive *npya* expression was found in a cluster of dorsal tegmentum nucleus (DTN), potentially near the Edinger–Westphal nucleus (EW, Figure 1j(1–3)). Two rhombencephalic *npya* expressions were found, one in a small cell cluster located lateral to the fasciculus longitudinalis medialis (FLM, Figure 1k(1–2)), and a larger cluster located ventral to the Ve4 close to the trigeminal nerve (nV, Figure 1l(1–2)).

#### 3.1.2 npyb

**FIGURE 2:**
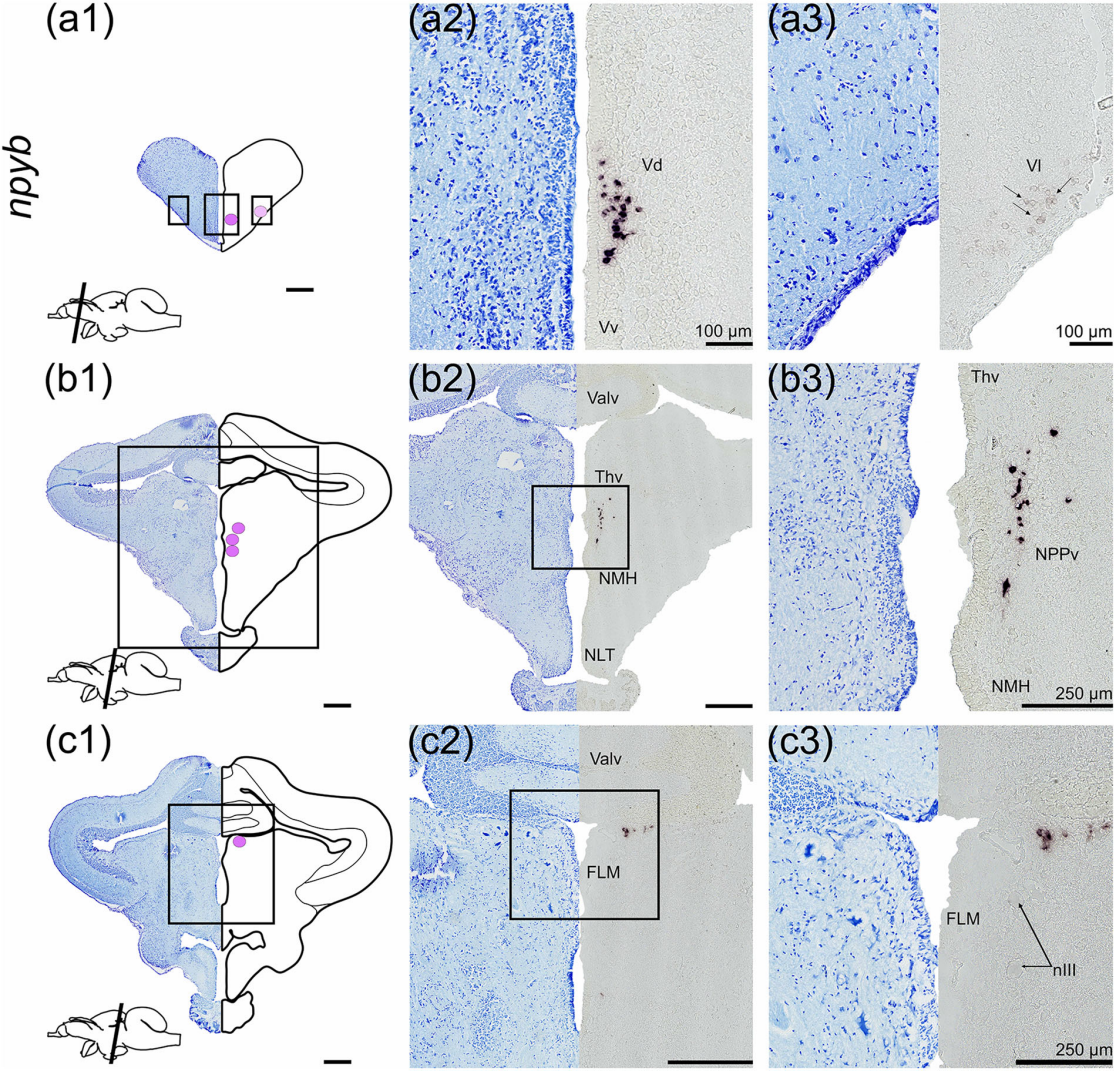
*npyb* mRNA expression in Atlantic salmon parr brain from rostral (a) to caudal (c) brain regions. (a1–c1) Nissl‐staining and schematic drawing illustrating *npyb* expression by purple dots. Lower left corner represents a schematic drawing of the salmon brain indicating the position of the section (a2–c2, a3–c3). Nissl‐staining and corresponding *npyb* expression with neuroanatomical structures. (a) *npyb* expression in the Vd, Vv and Vl parts of ventral telencephalon. (b) *npyb* expression near the Thv and NPPv. (c) *npyb* expression near the FLM and nIII. Abbreviations: FLM, fasciculus longitudinalis medialis; nIII, nervus oculomotorius; NMH, nucleus magnocellularis hypothalami; NPPv, nucleus posterioris periventricularis; Thv, ventral thalamus; Valv, valvula cerebelli; Vd, dorsal nucleus of ventral telencephalon; Vl, lateral nucleus of ventral telencephalon; Vv, ventral nucleus of ventral telencephalon. Scale bar (if no other indication) = 500 μm

*npyb* was detected in the telencephalon and diencephalon brain regions (Figure 2). In the telencephalon, *npyb* mRNA expression was found in the dorsal nucleus of the ventral telencephalon (Vd) toward the telencephalic ventricle in the mid telencephalon (rostral‐caudal direction) about 600 μm (Figure 2a(1–2)). Light staining for *npyb* was found in a few cells of the lateral nucleus of ventral telencephalon (Vl, Figure 2a(1–3)). Ventral to the optic tectum, one *npyb* expressing cell cluster was observed in the ventral thalamus region (Thv) (Figure 2b(1–3)) toward the hypothalamic NMH near the nucleus posterioris periventricularis (NPPv, Figure 2(b3)). A cell cluster expressing *npyb* was observed just ventral to the cerebellar valvula adjacent to the medial longitudinal fasciculus (FLM, Figure 2c(1–3)).

### 3.2 agouti‐related peptide 1 (agrp1)

**FIGURE 3:**
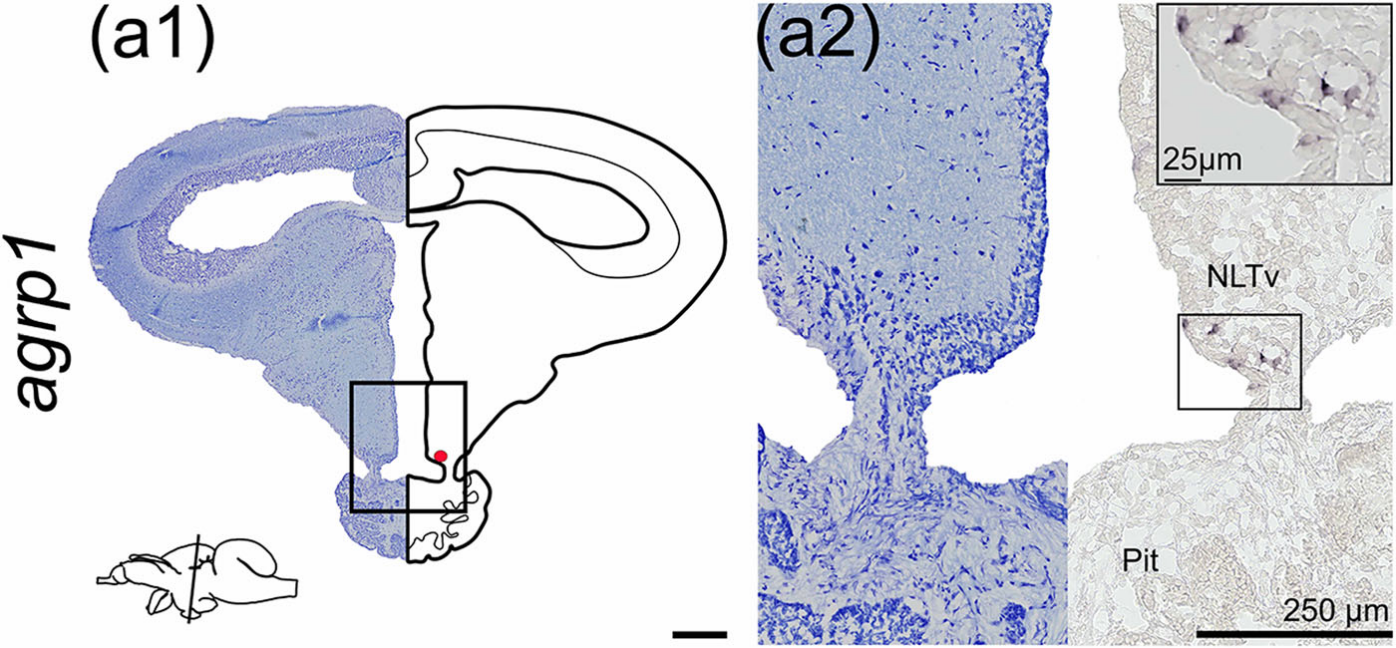
*agrp1* mRNA expression in Atlantic salmon parr brain. (a1) Nissl‐staining at equivalent level to the schematic drawing illustrating *agrp1* expression by red dot. Lower left corner represents a schematic brain indicating the position of the section. (a2) Nissl‐staining and corresponding *agrp1* expression in the ventral nucleus lateralis tuberis (NLTv) at the pituitary stalk. Abbreviation: Pit, pituitary. Scale bar (if no other indication) = 500 μm

Analysis by ISH of *agrp1* showed labeled neurons in the hypothalamic NLT, including the ventral NLT (NLTv, Figure 3). *agrp1*‐expressing neurons located at the pituitary stalk were situated medially toward the infundibulum as infundibular cerebrospinal‐fluid contacting cells, and a few neurons laterally from cerebrospinal fluid, connecting the posterior pituitary and caudal hypothalamus. *agrp1* mRNA expression was not detected in any other brain region.

### 3.3 agouti‐related peptide 2 (agrp2)

**FIGURE 4:**
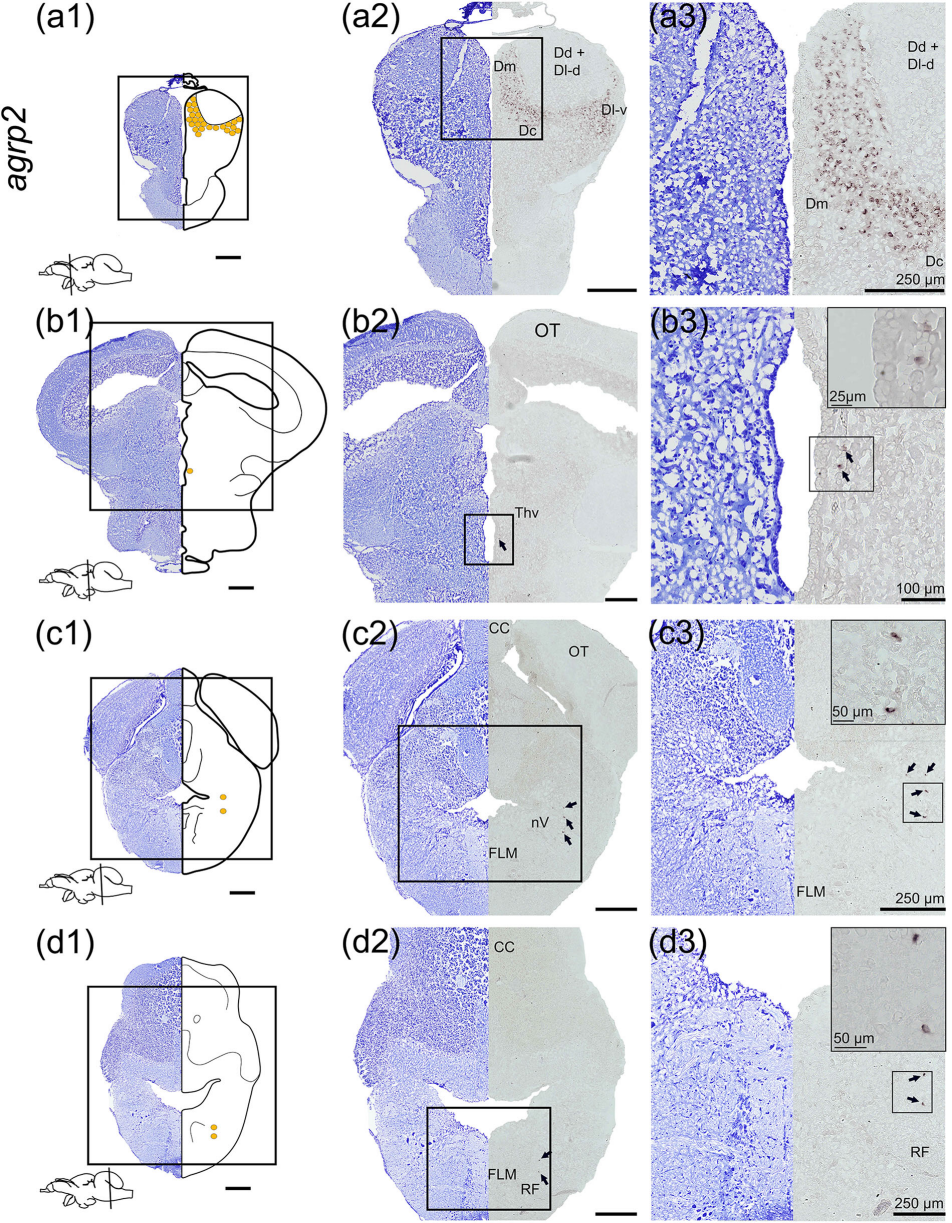
*agrp2* mRNA expression in Atlantic salmon parr brain. (a1–d1) Nissl‐staining compared to schematic drawing illustrating *agrp2* expression by yellow dots. Lower left corner represents a schematic brain indicating the position of the section. (a2–d2, a3–d3) Nissl‐staining and corresponding *agrp2* expression with neuron anatomical structures. (a) *agrp2* expression in the Dm, Dc, and Dl‐v parts of the telencephalon. (b) *agrp2* expression in the Thv. (c) *agrp2* expression near the FLM and (d) the RF. Abbreviations: Dc, central zone of dorsal telencephalon; Dd, dorsal part of dorsal telencephalon; Dl‐d, dorsal part of lateral zone of dorsal telencephalon; Dl‐v, ventral part of lateral zone of dorsal telencephalon; Dm, medial zone of dorsal telencephalon; FLM, fasciculus longitudinalis medialis; OT, optic tectum; RF, reticular formation; Thv, ventral thalamus. Scale bar (if no other indication) = 500 μm

*agrp2* was mainly expressed in the telencephalon, but a scattered expression was also found in the diencephalon and rhombencephalon (Figure 4). In the telencephalon, neurons expressing *agrp2* demonstrated a specific pattern from the medial region (Dm) and central region (Dc) toward the ventral part of the lateral zone (Dl‐v) of the dorsal telencephalon (Figure 4a(1–3)). A few *agpr2*‐positive neurons were located in the ventral thalamus (Thv, Figure 4b(1–3)). In the rhombencephalon, *agrp2* mRNA expression was identified in cells lateroventral to the rhombencephalic ventricle in small nuclei near large nuclei of the nucleus motorius nervi trigemini (nV, Figure 4c(1–3)) and laterally to the FLM in the reticular formation (RF, Figure 4d(1–3)).

### 3.4 cocaine‐ and amphetamine‐regulated transcript (cart)

The *cart* paralogs mRNA distributions in the brain of Atlantic salmon parr were analyzed by ISH using seven distinct RNA probes (Table 1). The results are presented in a rostrocaudal direction from the most abundant, *cart2b*, to the lowest abundant *cart* paralogs (*cart4*, *1a*, and *1b*).

#### 3.4.1 cart2b

*cart2b* was the most abundant *cart* paralog and showed a wide distribution in several brain regions (Figure 5), being continuously observed from the olfactory bulb to the thalamus. In the olfactory bulb, numerous cells expressing *cart2b* were found in the granular cell layer (strg, Figure 5b(1–2)). *cart2b‐*positive cells were found in the subpallium in the dorsal nucleus of ventral telencephalon (Vd) near the telencephalic ventricle (Figure 5c(1–3)). In the lateral telencephalon, a cluster of cells expressing *cart2b* was observed in the lateral nucleus of the ventral telencephalon (Vl). Scattered cells expressing *cart2b* were observed in the ventral nucleus of ventral telencephalon (Vv), lateral (Dl‐d and Dl‐v), medial (Dm), and dorsal (Dd) zone of the dorsal telencephalon (Figure 5(c3)). The *cart2b* mRNA expression from strg and Vd could be followed continuously to the periventricular preoptic region, as shown in nucleus posterior periventricularis (Ppp, Figure 5d(1–3)). *cart2b* was detected from the optic tectum in the periventricular layer (SPV) toward the stratum album centrale (SAC) border (Figure 5(e2)), and from the dorsal‐most region adjacent to torus longitudinalis until the base of the optic tectum near torus semicircularis (Figure 5(e1) (f1) (g1) (h1), and (i1)). In the midbrain, *cart2b* was present in the dorsal thalamus (Thd), posterior tuberculum (Pt) toward the diencephalic ventricle, and in the hypothalamus (Figure 5e,h).

FIGURE 5
*cart2b* mRNA expression neurons in Atlantic salmon parr brain. (a) Schematic representation of the brain indicating the position of each transverse section. (b1–l1) Nissl‐staining compared to schematic drawing illustrating *cart2b* expression by blue dots. (b2‐i2, c3‐i3) Nissl‐staining and corresponding *cart2b* expression along with neuroanatomical structures. (b) *cart2b* expression in the olfactory bulb stgr. (c) *cart2b* expression in the Dm, Dd, and Dl zones of dorsal telencephalon as well as the Vd, Vl and Vv nucleus of ventral telencephalon. (d) *cart2b* expression in the preoptic region—Ppp. (e) *cart2b* expression in the SPV, Thd, Pt, NAT, and NLTv. (f) *cart2b* expression in the SPV, Thd, NMH, NAT, tlat, and Lih. (g) *cart2b* expression in the SPV, scattered neurons in the Ts, large cluster near NMH and PVO, scattered neurons in NAT, nrl, Ce, tlat, and Lih. (h) *cart2b* expression in the SPV, and dorsal midbrain tegmentum toward Ts and the nlv, in the NDILm and nrl, and SV. (i) *cart2b* expression in the SPV, gran of the cerebellum, and in the lcoer and rets. Abbreviations: Ce, nucleus centralis lobi inferioris hypothalamic; Cho, optic chiasm; D, dorsal telencephalon; Dd, dorsal zone of dorsal telencephalon; Dl‐d, dorsal part of lateral zone of dorsal telencephalon; Dl‐v, ventral part of lateral zone of dorsal telencephalon; Dm, medial zone of dorsal telencephalon; Ggl, stratum ganglionare—cerebelli; Gran, stratum granulare—cerebelli; Hab, habenula; Lcoer, locus coeruleus; Lih, lobus inferior hypothalami; Mcba, tractus mesencephalo‐cerebellaris anterior; Mol, stratum moleculare—cerebelli; NAT, nucleus anterior tuberis; NDILm, medial part of the diffuse nucleus of inferior lobe; NLT, nucleus lateralis tuberis; NLTv, ventral nucleus lateralis tuberis; NLV, nucleus lateralis valvulae; NMH, nucleus magnocellularis hypothalami; NRL, nucleus recessi lateralis; OT, optic tectum; Ppp, posterior parvocellular preoptic nucleus; Pt, posterior tuberculum; PVO, paraventricular organ; Rets, formatio reticularis pars superior; SAC, stratum album centrale; SPV, stratum periventriculare of the optic tectum; Stgr, stratum granulare—bulbi olfactory; SV, saccus vasculosus; Thd, dorsal thalamus; Thv, ventral thalamus; Tlat, torus lateralis; Toll, tractus olfactorius lateralis; Ts, torus semicircularis; Valv, Valvula cerebelli; Vd, dorsal nucleus of ventral telencephalon; Vl, lateral nucleus of ventral telencephalon; Vv, ventral nucleus of ventral telencephalon; Scale bar (if no other indication) = 500 μm

**Figure:**
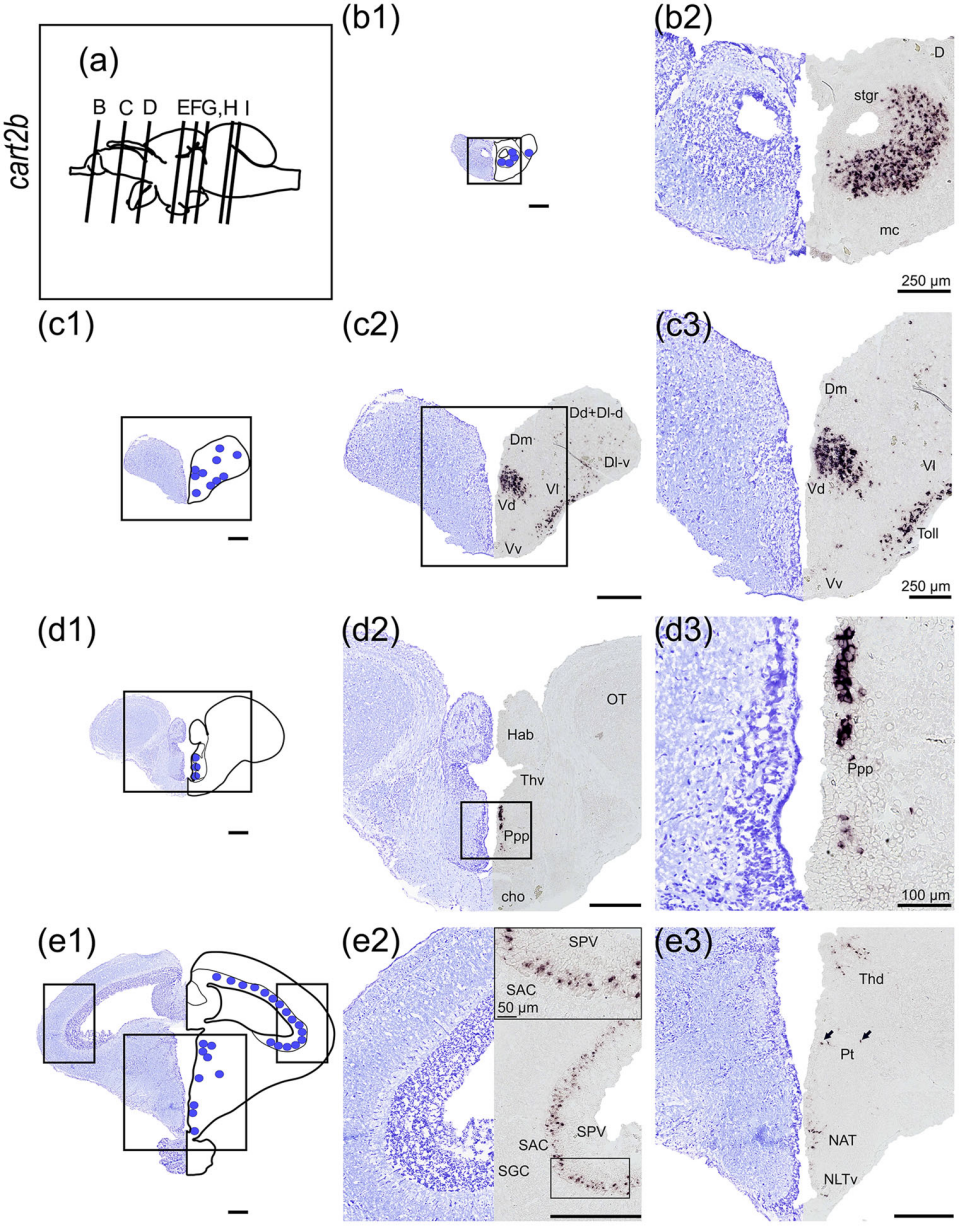

**Figure:**
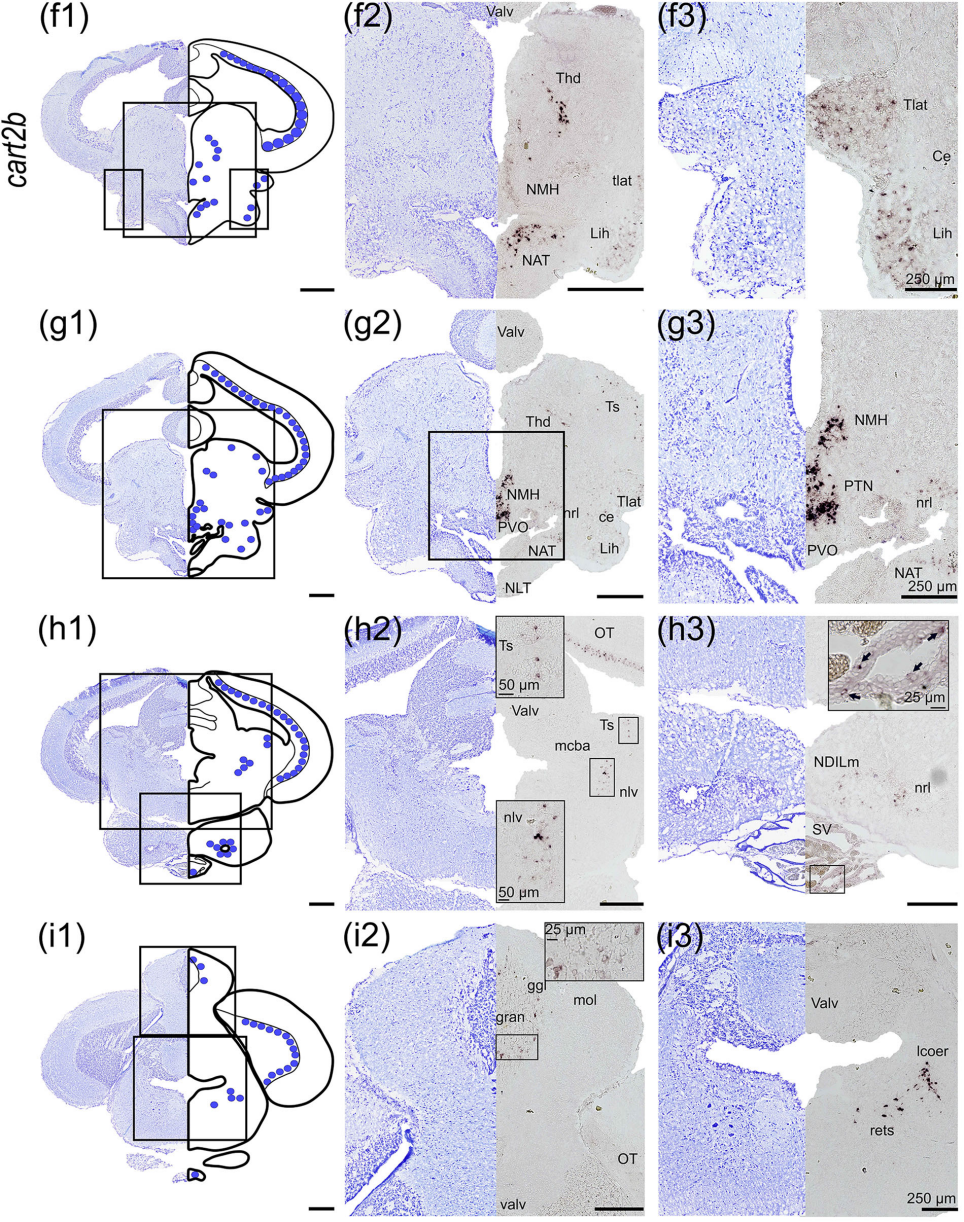

*cart2b* mRNA was abundant in the hypothalamus, in a dorsoventral direction from the NAT toward the ventral NLT (NLTv, Figure 5(e3)). Scattered cells expressing *cart2b* were observed laterally in *torus lateralis* (tlat) and *lobus inferior hypothalami* (Lih) toward the cerebrospinal fluid (Figure 5(f3)). A cluster of cells expressing abundantly *cart2b* were also observed in the paraventricular organ (PVO), nucleus posterior tuberis (PTN) as well as in NMH (Figure 5g(1–3)). From NAT, *cart2b* expression continued to be detected dorsolateral to the infundibulum, into nucleus recessi lateralis (NRL) and medial part of the diffuse nucleus of Lih (NDILm, Figure 5(h1) and (h3)). Scattered cells expressing *cart2b* were observed in *saccus vasculosus* (SV, Figure 5(h3)).

Ventral to the valvula in dorsal tegmentum, scattered neurons expressing *cart2b* were observed in torus semicircularis (Ts) toward nucleus lateralis valvulae (nlv, Figure 5(g2) and (h2)). In the rhombencephalon, *cart2b* was observed in stratum ganglionare (ggl) of corpus cerebelli (Figure 5i(1–2)) and ventrolateral to the fourth ventricle near locus coeruleus (lcoer) and formatio reticularis pars superior (rets, Figure 5(i1) and (i3)).

#### 3.4.2 cart3a

*cart3a* mRNA expression was identified in the telencephalon, midbrain, and rhombencephalon (Figure 6). In the telencephalon, neurons expressing *cart3a* were identified in the ventral nucleus of ventral telencephalon (Vv, Figure 6b(1–b2)), in the ventrolateral telencephalon in nucleus entopeduncularis (ent), and within SOC of the preoptic region (Figure 6c(1–3)). The *cart3a* in the optic tectum was expressed in the less densely populated neurons in the torus longitudinalis (TL) toward the white matter of the torus longitudinalis (TLw, Figure 6(d1) (d2) (e1), and (e2)). *cart3a* expression was observed in scattered cells in the dorsal thalamus (Figure 6(d3)), and in the hypothalamus dorsal to the paraventricular organ (PVO) in PTN and NMH (Figure 6(e3)). *cart3a* expression was also found in the dorsal mesencephalic tegmentum (DTN)—possibly near the EW (Figure 6f(1–3)), dorsomedial to fasciculi longitudinalis medialis (FLM, Figure 6g(1–2)), and scattered neurons laterally to the rhombencephalic ventricle near lcoer (Figure 6(g3)). In rostral rhombencephalon, *cart3a* mRNA was found ventral of nervus trigeminus (nV) in nervus motorius nervi trigemini (nVm, Figure 6h(1–3)).

FIGURE 6
*cart3a* mRNA expression in Atlantic salmon parr brain. (a) Schematic representation of the brain indicating the position of each transverse section. (b1–h1) Nissl‐staining compared to schematic drawing illustrating *cart3a* expression by red dots. (b2–h2, c3–h3) Nissl‐staining and corresponding *cart3a* expression along with neuroanatomical structures. (b) *cart3a* expression in the Vv. (c) *cart3a* expression in the ent and in the SOC. (d) *cart3a* expression in the TLw of the optic tectum and in the Thd. (e) *cart3a* expression in the TL, and in the NMH and PVO. (f) *cart3a* expression in the TL and dorsal tegmentum near EW. (g) *cart3a* expression near the FLM and lcoer. (h) *cart3a* expression near the nV and nVm. Abbreviations: Cho, optic chiasm; cp, commissura posterior; Ent, nucleus entopeduncularis; EW, Edinger–Westphal nucleus; FLM, fasciculus longitudinalis medialis; fMth, fiber of Mauthner cell; Lcoer, locus coeruleus; NAT, nucleus anterior tuberis; NMH, nucleus magnocellularis hypothalami; nV, nervi trigemini; nVm, nucleus motorius nervi trigemini; OT, optic tectum; PVO, paraventricular organ; SOC, supraoptic/suprachiasmatic nucleus; Thd, dorsal thalamus; TL, torus longitudinalis; TLw, white matter of the torus longitudinalis; Valv, valvula cerebelli; Vv, ventral nucleus of ventral telencephalon. Scale bar (if no other indication) = 500 μm

**Figure:**
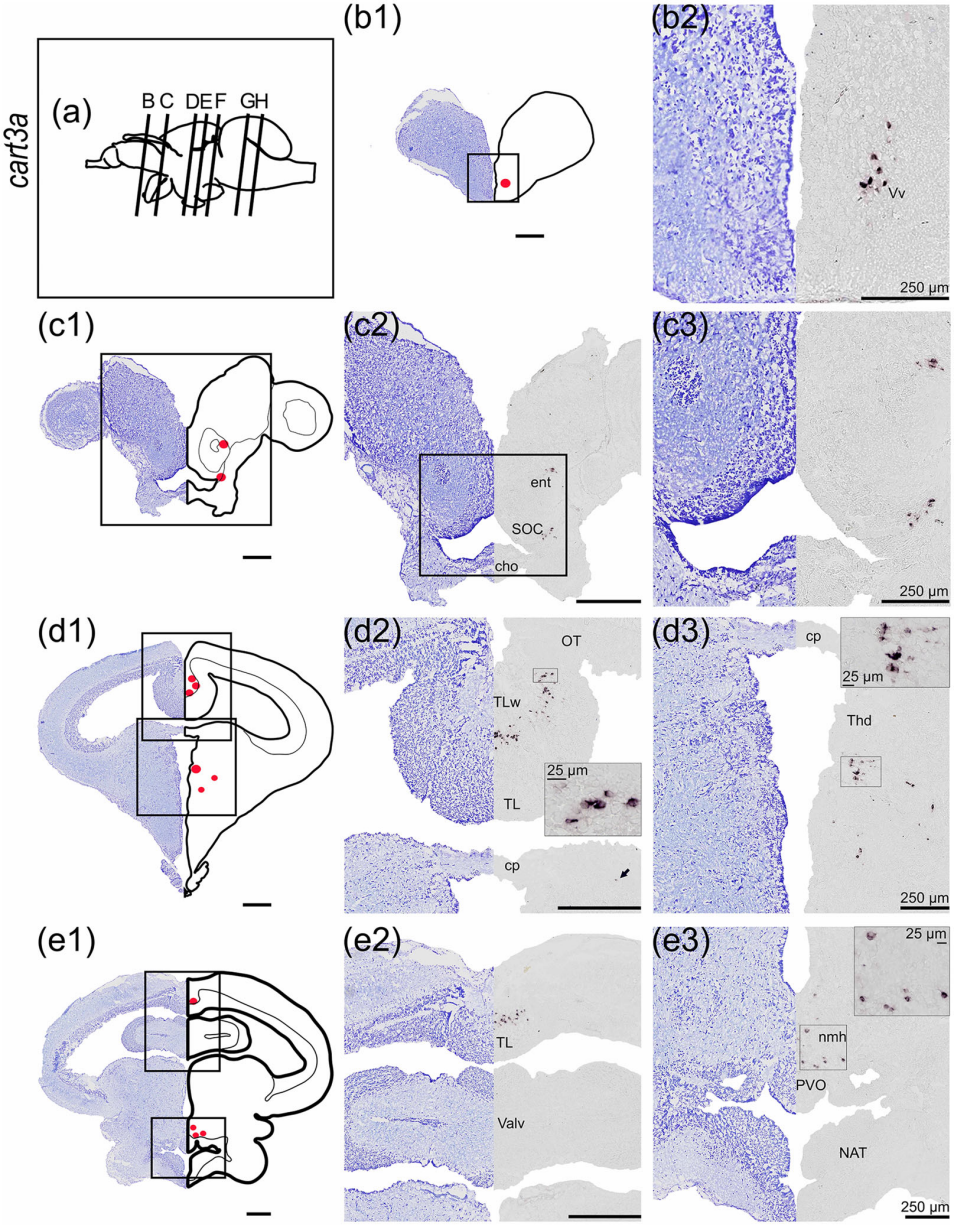

**Figure:**
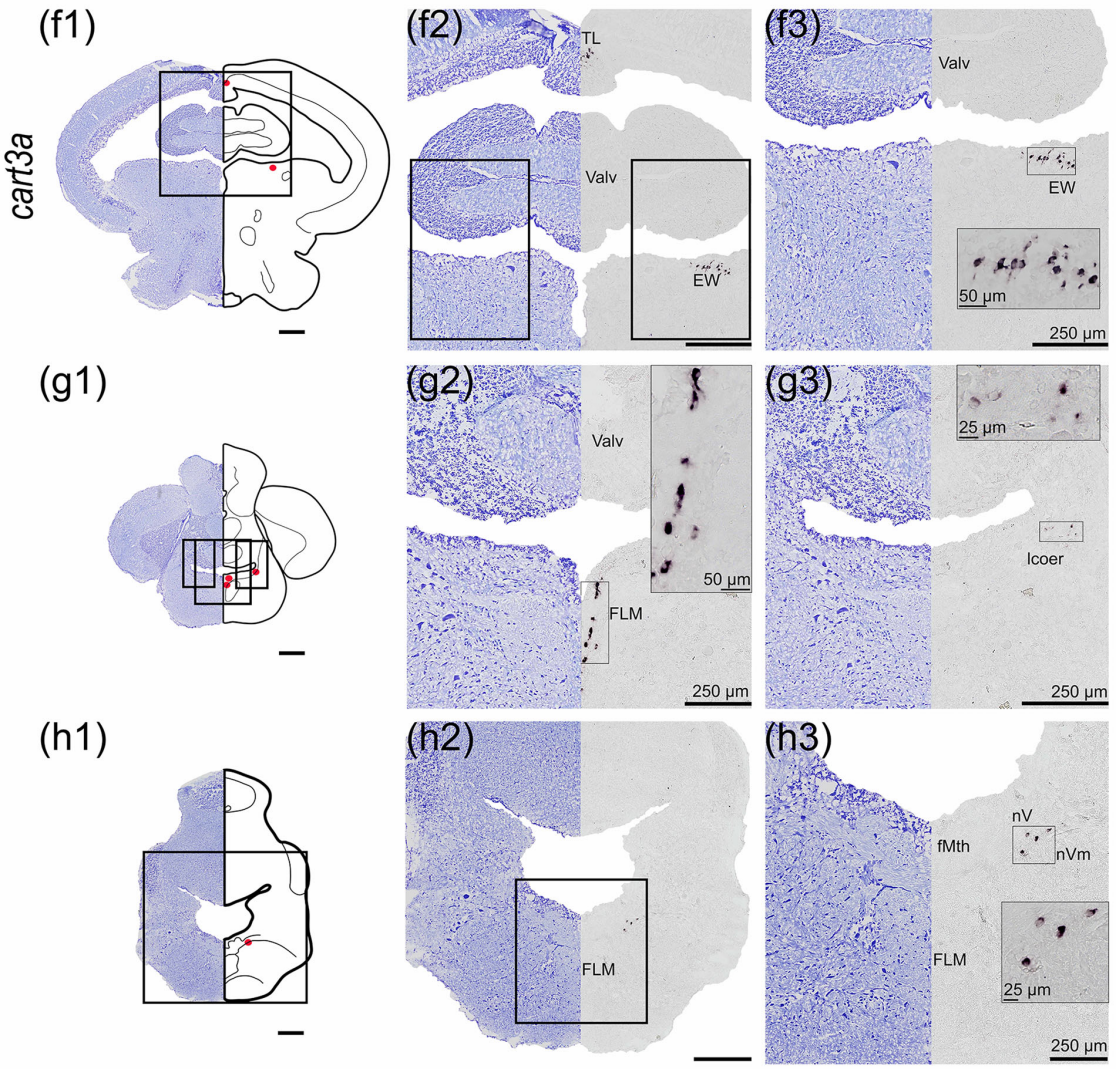

#### 3.4.3 cart3b

The localization of *cart3b* by ISH demonstrated its presence in the preoptic region, thalamus, tegmentum, and rhombencephalon (Figure 7). *cart3b* mRNA expression was detected in the caudal preoptic region, specifically in the posterior part of the parvocellular preoptic nucleus (Ppp, Figure 7b(1–2)). Ventral to the optic tectum in the diencephalon, *cart3b* probe labeled a neuronal line from the dorsal thalamus (Thd) to the ventrolateral direction of the hypothalamic *torus lateralis* (tlat, Figure 7c(1–3)). Ventral to the valvula, *cart3b* was expressed in scattered neurons of the central and ventral torus semicircularis (TS, Figure 7d(1–3)). The *cart3b* probe also hybridized scattered neurons located ventrolateral to the FLM (Figure 7e(1–3)), neurons near nervus trigemini (nV, Figure 7f(1–3)), and neurons in the formatio reticularis pars superior (rets) as well as dorsal cells to rets (Figure 7g(1–3)).

FIGURE 7
*cart3b* mRNA expression in Atlantic salmon parr brain. (a) Schematic representation of the brain indicating the position of each transverse section. (b1–g1) Nissl‐staining compared to schematic drawing illustrating *cart3b* expression by pink dots. (b2–g2, c3–g3) Nissl‐staining and corresponding *cart3b* expression along with neuroanatomical structures. (b) *cart3b* expression in the preoptic region—Ppp. (c) *cart3b* expression in a ventrolateral direction from the Thd toward tlat. (d) *cart3b* expression in dorsal tegmentum near Ts. (e) *cart3b* expression ventral to the FLM. (f) *cart3b* expression near nV. (g) *cart3b* expression ventral to the FLM near the rets. Abbreviations: FLM, fasciculus longitudinalis medialis; fMth, fiber of Mauthner cell; Lih, lobus inferior hypothalami; nV, nervi trigemini; ppp, posterior parvocellular preoptic nucleus; Rets, formatio reticularis pars superior; Tbc, tractus tecto‐bulbaris cruciatus; Thd, dorsal thalamus; Tlat, torus lateralis; Ts, torus semicircularis; Valv, valvula cerebelli; Ve4, fourth ventricle (rhombencephali). Scale bar (if no other indication) = 500 μm

**Figure:**
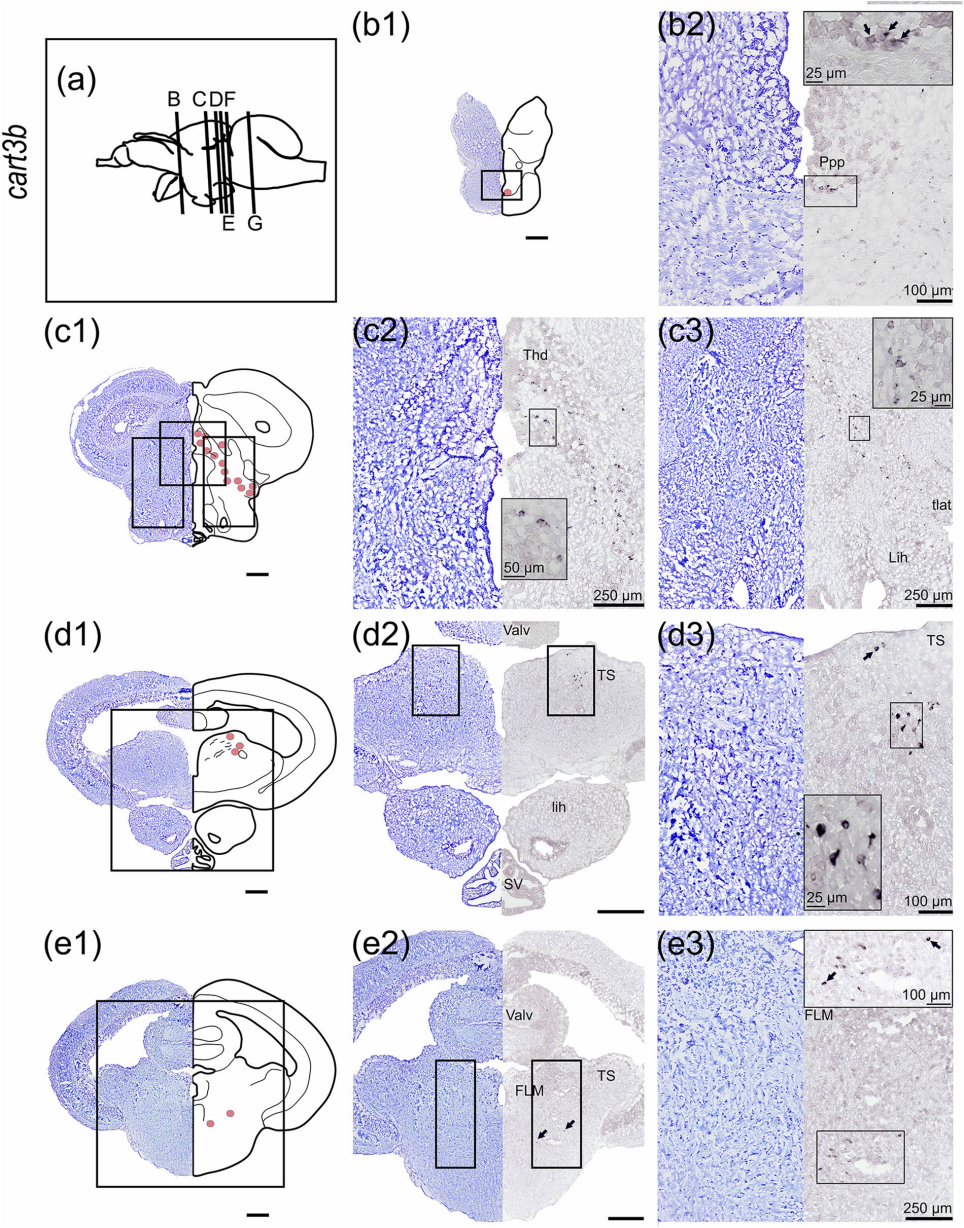

**Figure:**
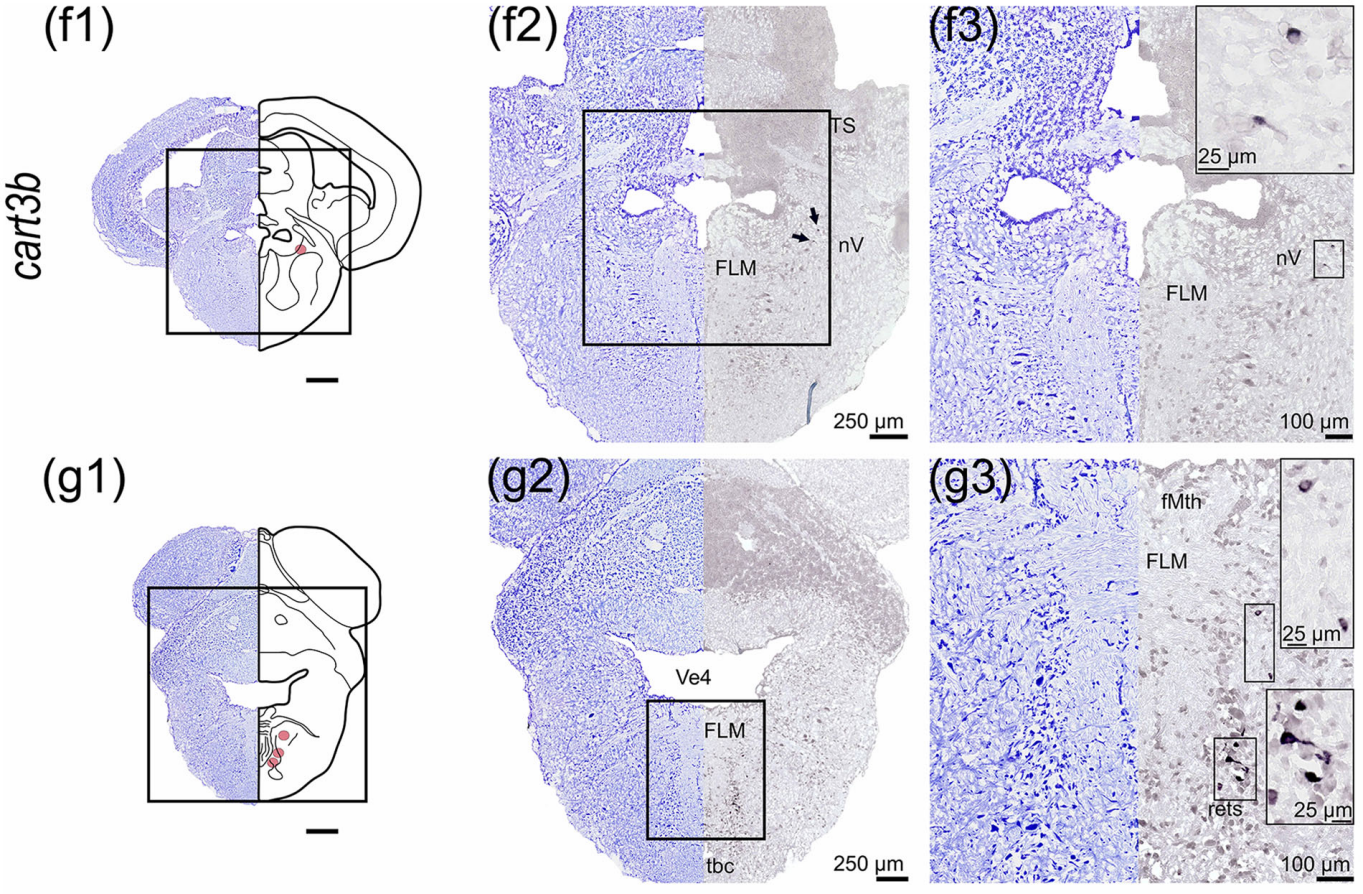

#### 3.4.4 cart2a

**FIGURE 8:**
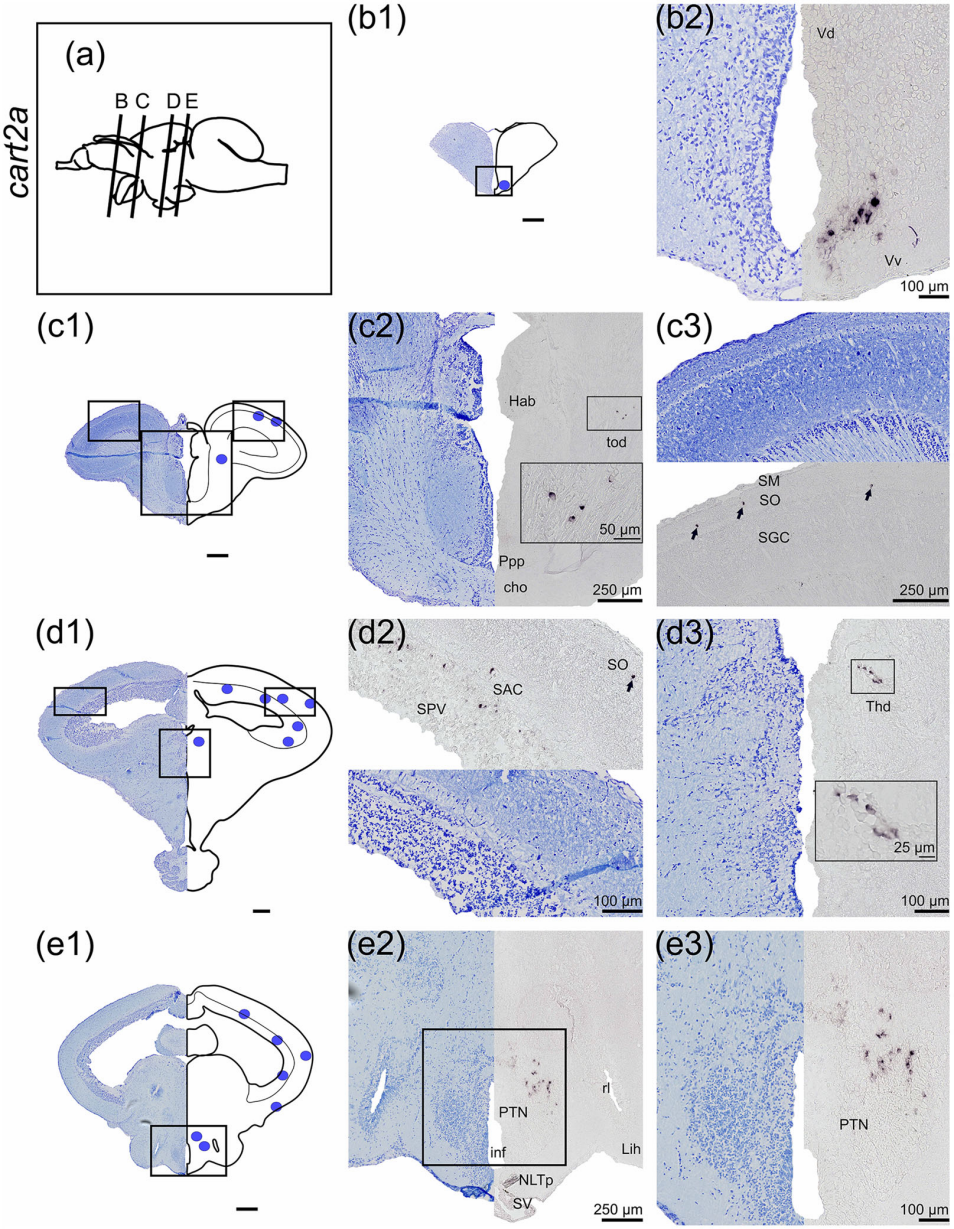
*cart2a* mRNA expression in Atlantic salmon parr brain. (a) Schematic representation of the brain indicating the position of each transverse section. (b1–e1) Nissl‐staining compared to schematic drawing illustrating *cart2a* expression by blue dots. (b2‐e2, c3‐e3) Nissl‐staining and corresponding *cart2a* expression along with neuroanatomical structures. (b) *cart2a* expression in Vv of telencephalon. (c) *cart2a* expression in tod and SO. (d) *cart2a* expression in SPV, SAC, and SO of the optic tectum and ventrally in the Thd. (e) *cart2a* expression in the hypothalamus near the PTN. Abbreviations: Cho, optic chiasm; Hab, habenula; inf, infundibulum; Lih, lobus inferior hypothalamic; NLTp, posterior nucleus lateralis tuberis; Ppp, posterior parvocellular preoptic nucleus; PTN, nucleus posterior tuberis; Rl, recessi lateralis; SAC, stratum album centrale (tecti mesencephali); SM, stratum marginale (tecti mesencephali); SO, stratum opticum (tecti mesencephali); SPV, stratum periventriculare of the optic tectum; SV, saccus vasculosus; Thd, dorsal thalamus; Tod, tractus opticus dorsalis; Vd, dorsal nucleus of ventral telencephalon; Vv, ventral nucleus of ventral telencephalon. Scale bar (if no other indication) = 500 μm

*cart2a*‐expressing cells were identified in the telencephalon, optic tectum, thalamus, and hypothalamus (Figure 8). One *cart2a* neuronal cluster was present in the ventral nucleus of the ventral telencephalon (Vv) toward the telencephalic ventricle (Figure 8b(1–2)). Scattered *cart2a*‐positive cells were detected in tractus opticus pars distalis (tod, Figure 8c(1–2)). In the optic tectum, scattered cells expressing *cart2a* were observed in stratum marginale (SM), stratum opticum (SO, Figure 8(c3)), in the album layer (SAC), and evenly distributed in the periventricular layer (SPV) toward the SAC (Figure 8d(1–2) and (e1)). In the midbrain, one *cart2a* cell cluster was observed in the dorsal thalamus (Thd, Figure 8d(1–3)). In the hypothalamus, a cluster of cells expressing *cart2a* was identified in the PTN (Figure 8e(1–3)).

#### 3.4.5 *cart4*, *1b*, and *1a*

**FIGURE 9:**
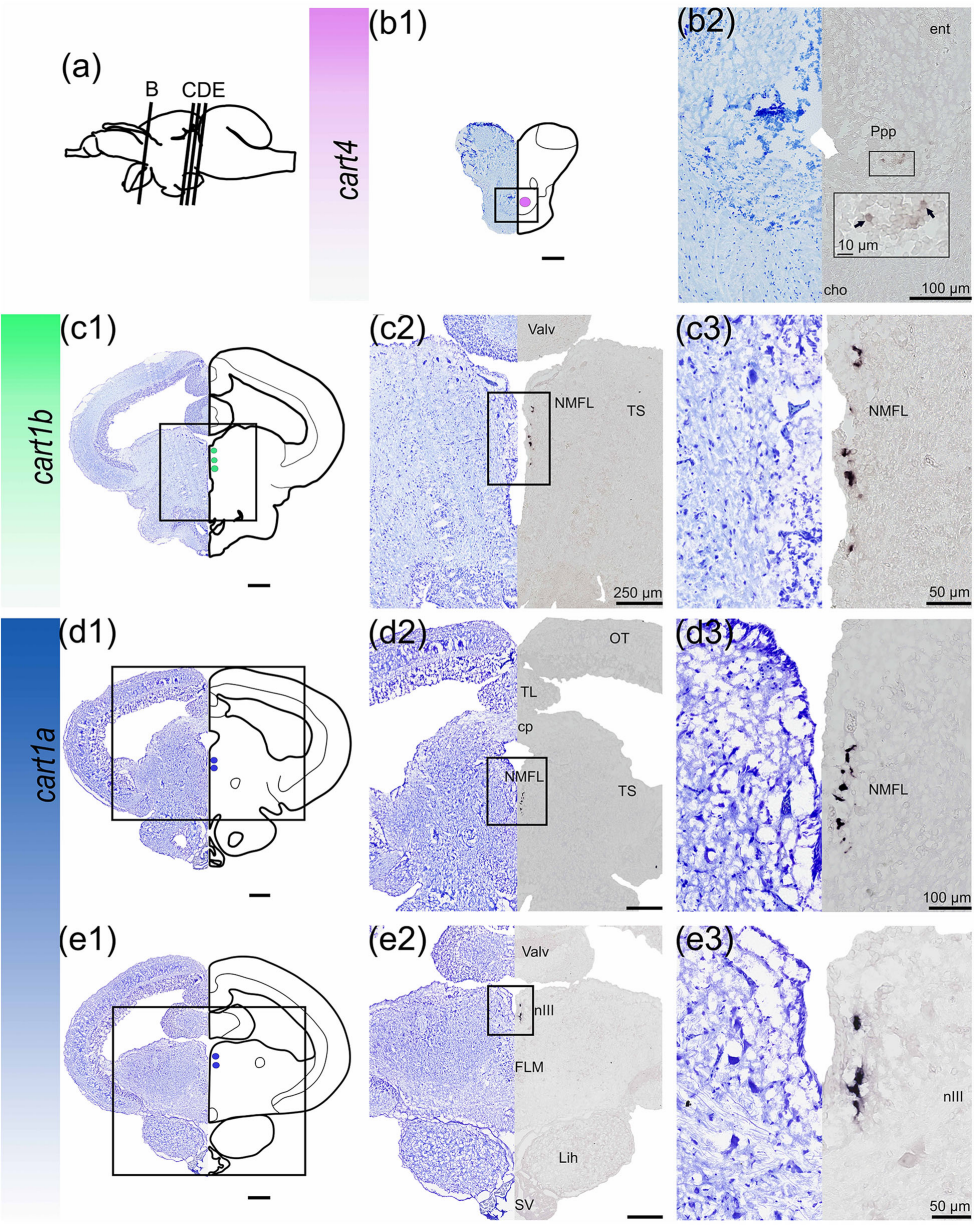
*cart4*, *1b*, and *1a* mRNA expression in Atlantic salmon parr brain. (a) schematic representation of the brain indicating the position of each transverse section. (b1–e1) Nissl‐staining compared to schematic drawing illustrating *cart4* expression by pink dot, *cart1b* by green dots, and *cart1a* by blue dots. (b2–e2, c3–e3) Nissl‐staining and corresponding *cart4*, *1b*, and *1a* expression along with neuroanatomical structures. (b) *cart4* expression the preoptic region—Ppp. (c) *cart1b* expression near the NMFL. (d) *cart1a* expression near the NMFL. (e) *cart2a* expression near the nIII. Abbreviations: Cho, optic chiasm; Cp, commissura posterior; Ent, nucleus entopeduncularis; FLM, medial longitudinal fasciculus; Lih, inferior hypothalamic lobe; nIII, Nucleus oculomotorius; NMFL, nucleus medial longitudinal fasciculus; OT, optic tectum; Ppp, posterior parvocellular preoptic nucleus; SV, saccus vasculosus; TL, torus longitudinalis; Ts, torus semicircularis; Valv, valvula cerebelli. Scale bar (if no other indication) = 500 μm

*cart4*, *1b*, and *1a* were expressed in distinct brain regions from the rostral to the caudal direction (Figure 9). *cart4* was only expressed in the most rostral area of the diencephalon, in the posterior parvocellular preoptic nucleus (Ppp, Figure 9b(1–2)). In the dorsomedial mesencephalic tegmentum ventral to the valvula near the nucleus medial longitudinal fasciculus (NMFL), *cart1a* (Figure 9c(1–3)) and *cart1b* (Figure 9d(1–3)) mRNA expression were identified. *cart1b* was only observed in one cell cluster, while the *cart1a* probe identified two separate clusters of neurons adjacent and medial to the FLM and oculomotor nucleus (NIII, Figure 9e(1–3)).

### 3.5 pro‐opiomelanocortin (pomc)

**FIGURE 10:**
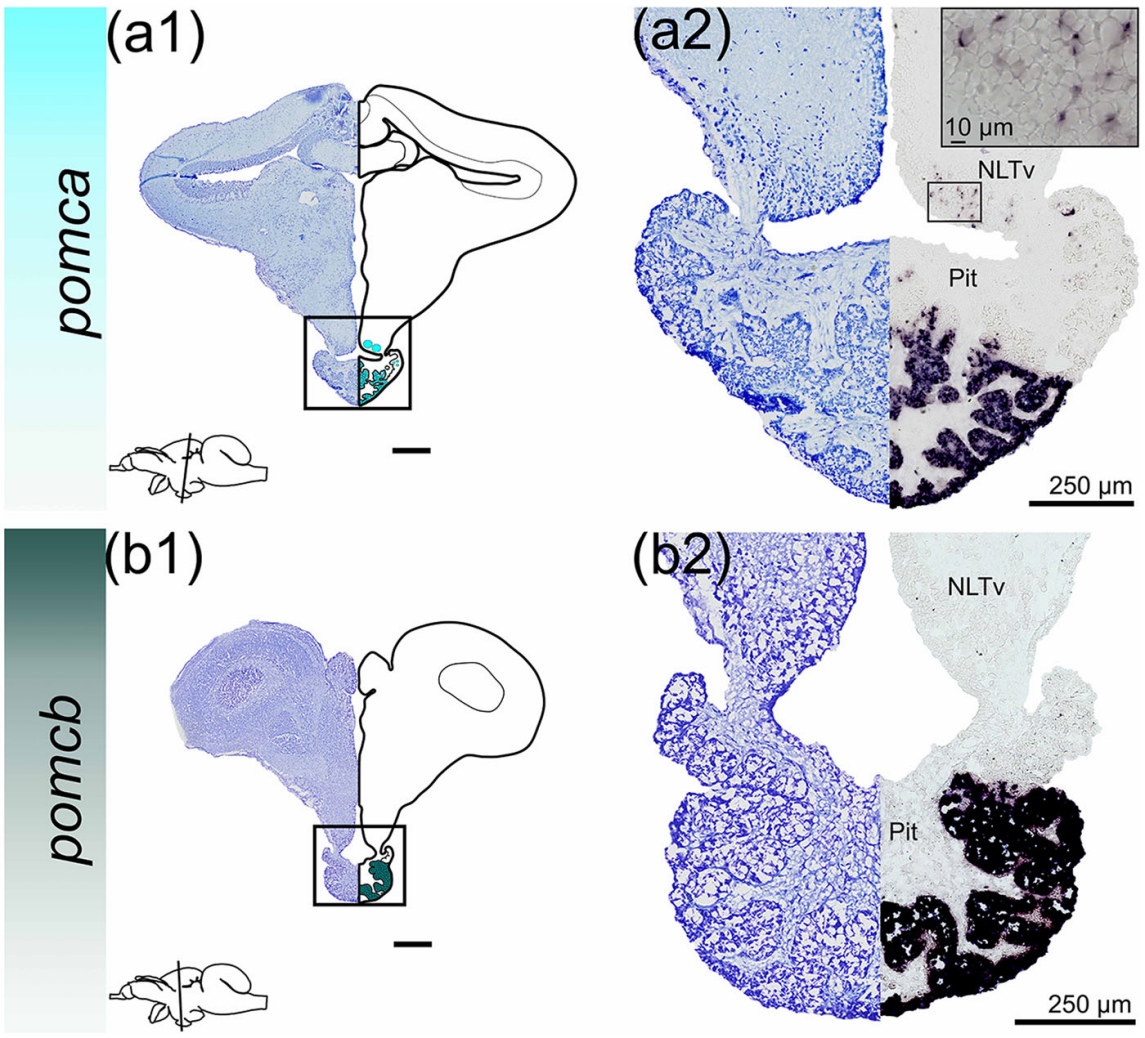
*pomc* mRNA expression in Atlantic salmon parr brain. (a1–b1) Nissl‐staining compared to schematic drawing illustrating *pomca* expression by light blue dots, and *pomcb* expression by green dots. Lower left corner represents a schematic brain indicating the position of the section. (a2–b2) Nissl‐staining and corresponding *pomca* and *pomcb* expression with neuroanatomical structures. (a) *pomca* expression in the NLTv and adenohypophysis of the Pit. (b) *pomcb* expression in the adenohypophysis of the Pit. Abbreviations: NLTv, ventral nucleus lateralis tuberis. Pit, pituitary. Scale bar (if no other indication) = 500 μm

In the Atlantic salmon parr brain, *pomca*‐expressing cells were detected in the pituitary (adenohypophysis), and in the NLTv of the hypothalamus (Figure 10a(1–2)). The hypothalamic *pomca‐*expressing cells were located medially toward the infundibulum. *pomcb* was strongly expressed in the adenohypophysis of the pituitary (Figure 10b(1–2)), and was not observed in the NLT, or in any other brain regions.

### 3.6 Hypothalamic expression of melanocortin system neuropeptides

**FIGURE 11:**
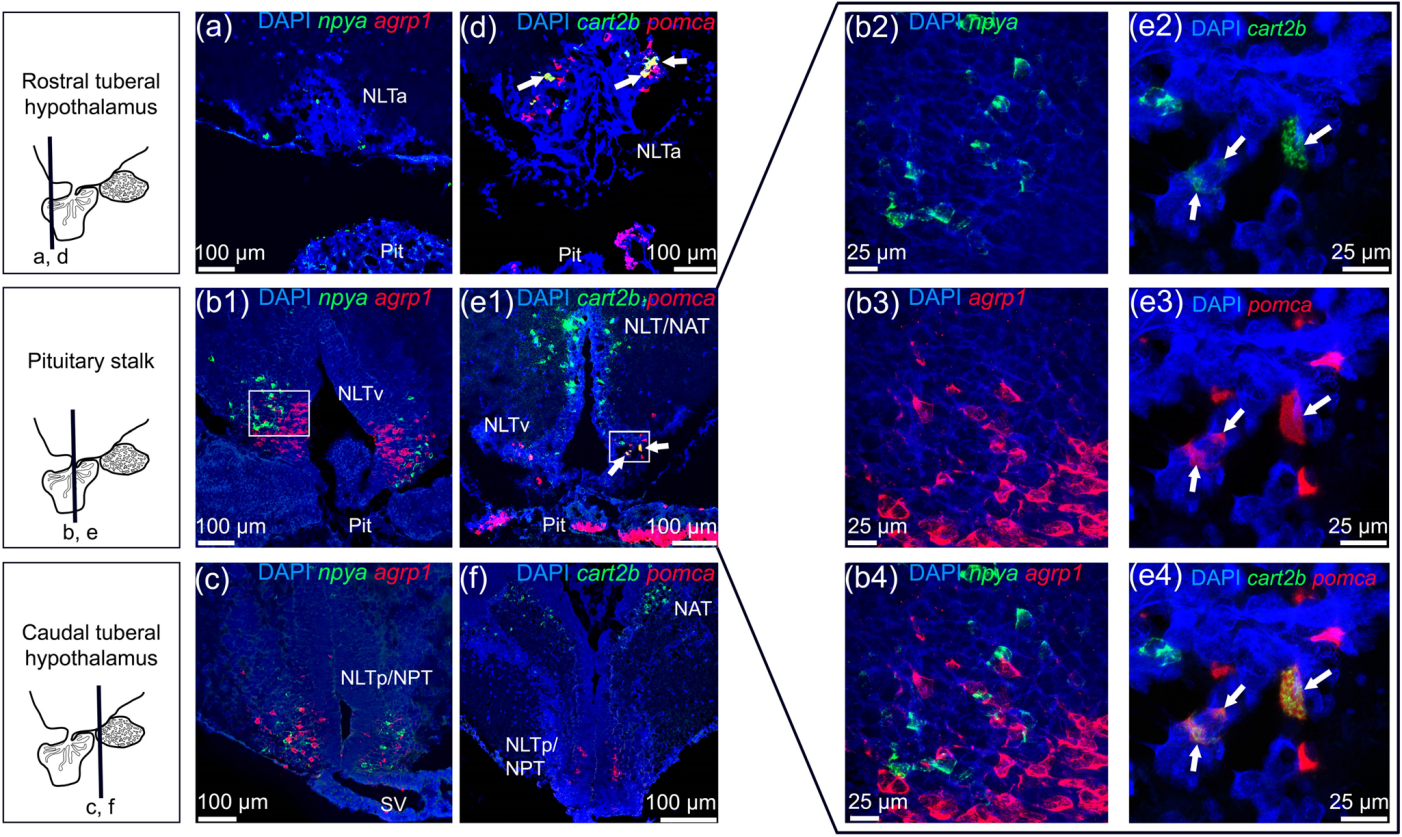
*npya*, *agrp1*, *cart2b*, and *pomca* mRNA expression in Atlantic salmon tuberal hypothalamus. Left side: schematic representation of the tuberal hypothalamus indicating the position of each transverse section. (a–c) *npya* (TSA‐green) and *agrp1* (FastBlue‐red) expression. (d–f) *cart2b* (TSA‐green) and *pomca* (FastRed‐red) expression. (a) *npya* expression in the NLTa of the rostral tuberal hypothalamus. (b1) *npya* and *agrp1* expression in the NLTv at the pituitary stalk. (b2–b4) *npya* and *agrp1* expression in neighboring neurons in the NLTv. The absence of yellow staining indicates no coexpression between *npya* and *agrp1* mRNA. (c) *npya* and *agrp1* expression in the NLTp/NPT of the caudal tuberal hypothalamus. (d) *cart2b* and *pomca* expression in the NLTa of the rostral tuberal hypothalamus. The presence of yellow staining (white arrows) indicates coexpression of *cart2b* and *pomca* mRNA. (e) *cart2b* and *pomca* expression in the NLTv of the tuberal hypothalamus. (e2–e4) The presence of yellow staining (white arrows) indicates coexpression of *cart2b* and *pomca* mRNA in the NLT. (f) *cart2b* expression in NAT, and *pomca* expression in the NLTp/NPT. Sections were mounted with ProLong Glass antifade medium with NucBlue (Invitrogen). Abbreviations: NAT, nucleus anterior tuberis; NLTa, anterior nucleus lateralis tuberis; NLTp, posterior nucleus lateralis tuberis; NLTv, ventral nucleus lateralis tuberis; NPT, nucleus posterior tuberis; Pit, pituitary; SV: saccus vasculosus

To determine whether the Atlantic salmon tuberal hypothalamus coexpress *npya*/*agrp1* and/or *cart2b*/*pomca*, double labeling fluorescent ISH was used. The results show that neurons expressing *npya* did not coexpress *agpr1* (Figure 11). In the anterior NLT (NLTa) of the rostral tuberal hypothalamus, few neurons expressed *npya* mRNA, but no *agrp1* expression was found in this region (Figure 11a). Toward the NLTv at the pituitary stalk, both *npya* and *agrp1* were abundantly expressed in neighboring neurons (Figure 11b(1–4)). *npya* and *agrp1* were still present in neighboring neurons of the ventral NLT (NLTv) bordering the nucleus posterior tuberis (NPT) in the caudal tuberal hypothalamus (Figure 11c), but their expression decreased, particularly for *agrp1*, in comparison to the NLTv at the pituitary stalk.

*cart2b*/*pomca* coexpression was observed in the NLTa and NLTv of the tuberal hypothalamus (Figure 11d,e). However, *cart2b* and *pomca* were mainly expressed in distinct neurons of the tuberal hypothalamus. The *cart2b*‐positive neurons were gradually located dorsally toward the NAT, while *pomca* expression remained ventrally in the NLT (Figure 11e,f). Thus, no *cart2b*/*pomca* coexpression was found in the caudal tuberal hypothalamus.

## 4 DISCUSSION

In this study, ISH was utilized to map the spatial distribution of *npy*, *agrp*, *cart*, and *pomc* in the Atlantic salmon parr brain (summarized in Table 2). The topology of these neuropeptides, particularly in the lateral tuberal nucleus (NLT), supports that the hypothalamic nucleus is associated with appetite and food intake regulation. We also demonstrated the presence of *cart2b/pomca* coexpression in the anterior and ventral NLT.

As a result of the salmonid‐specific fourth whole‐genome duplication event (Allendorf & Thorgaard, 1984; Lien et al., 2016), several paralogs of *npy*, *cart*, and *pomc* have been identified in Atlantic salmon (Kalananthan et al., 2021; Murashita et al., 2011; Tolås et al., 2021; Valen et al., 2011). Although the fate of the duplicated genes of key players in the melanocortin system are not yet fully understood, one hypothesis is that some genes will still play a role in appetite control by facilitating physiological, sensory, or periprandial responses. Indeed, our results demonstrate that these neuropeptides are expressed in the salmon brain regions known to be related to feeding and energy status. These regions include the hypothalamus, known to be related to regulation of vital homeostatic feeding control in both fish and mammals, as well as the olfactory bulb, telencephalon, optic tectum, and secondary gustatory nucleus, which are linked to feeding (Demski & Knigge, 1971; Volkoff et al., 2005). Indeed, we found *npy*, *agrp2*, and *cart* in the olfactory bulb, telencephalon, and optic tectum (Figures 1, 2, 3, 4, 5, 6, 7, 8, 9). Several of these brain regions are indirectly linked to chemical stimulation of appetite, either through inputs from sensory organs (olfaction and taste) or by hedonic (nonhomeostatic) regulation (Arikawa et al., 2020; Rossi & Stuber, 2018; Volkoff, 2019).

### 4.1 Hypothalamic expression of melanocortin system neuropeptides

The hypothalamic neuropeptides *npya*, *agrp1*, *cart2b*, and *pomca* are involved in appetite control as their expression levels responded to a fed/fasted state in Atlantic salmon (Kalananthan et al., 2021; Kalananthan, Murashita, et al., 2020; Murashita et al., 2011, 2009; Tolås et al., 2021; Valen et al., 2011). Here, we show the presence of these neuropeptides in the NLT region of the Atlantic salmon parr, the putative homolog to the mammalian arcuate nucleus ((Cerdá‐Reverter & Peter, 2003; Cerdá‐Reverter, Ringholm, et al., 2003) and reviewed in Biran et al. (2015)), supporting previous evidence that this region and these genes are involved in appetite control. This can be further supported by the presence of a few neurons coexpressing *cart2b/pomca*, and the expression of *agrp1* and *npya* in neighboring cells in the NLT (Figure 11). There is evidence that the homeostatic control of appetite by the melanocortin system involves the stimulation of hypothalamic arcuate nucleus first‐order orexigenic and anorexigenic neurons, which then project to second‐order hypothalamic neurons which in turn project to autonomic centers in the hindbrain (Morton et al., 2006; Schwartz et al., 2000). The resulting neuronal net output stimulates anabolic or catabolic pathways. Previous studies mapping the neuroanatomical distribution of melanocortin circuits in teleosts have hypothesized possible coexpressions (Delgado et al., 2017; Porter et al., 2017; Soengas et al., 2018); however, this has never been demonstrated. Thus, to our knowledge, this is the first evidence of coexpression between *cart2b/pomca* in the NLT region of a teleost species. In agreement with the findings of Jeong et al. (2018) in the hypothalamus of zebrafish, no coexpression of *npy/agrp1* was observed in Atlantic salmon NLT. Therefore, as previously suggested, coexpression of *npy/agrp1* might not be required for the action of these neuropeptides in appetite control of teleost fishes (Jeong et al., 2018). As a contrast, at least 90% of the neurons in the mammalian arcuate nucleus that express *Npy* or *Cart* also express *Agrp* or *Pomc*, respectively, and play a crucial role in a homeostatic regulation of appetite (Elias et al., 1998; Hahn et al., 1998; Schwartz et al., 2000). The limited number of neurons coexpressing *cart2b/pomca* in the tuberal hypothalamus of Atlantic salmon, and that there was no coexpression of *npya/agrp1* suggest that coexpression might not be required for homeostatic feeding control in Atlantic salmon and other teleost species.

The presence of *npy* in the NLT region seems to be conserved throughout evolution since it has been observed in several teleost species including sea bass (Cerdá‐Reverter et al., 2000), goldfish (Kojima et al., 2010), Atlantic cod (Le et al., 2016), and African cichlid fish (Porter et al., 2017). The NLT region is considered a site for integrating and releasing neurotransmitters to higher‐order neurons linked to neuroendocrine appetite control and feeding behavior (Rønnestad et al., 2017). In Atlantic salmon, *agrp1* was exclusively detected in the hypothalamic NLT (Figure 3). The NLT *agrp1* expression is in line with observations for other species like goldfish (Cerdá‐Reverter & Peter, 2003), zebrafish (Forlano & Cone, 2007; Koch et al., 2019; Shainer et al., 2017), sea bass (Agulleiro et al., 2014), African cichlid fish (Porter et al., 2017), and rainbow trout (Otero‐Rodino et al., 2019). Indeed, *agrp1* function has been associated with appetite control in Atlantic salmon (Kalananthan, Murashita, et al., 2020; Murashita et al., 2009). Furthermore, in zebrafish, it has been shown that *agrp*‐neurons are hypophysiotropic, projecting from the NLT to the pituitary (Zhang et al., 2012). The high degree of similarity in the NLT *agrp1* population among fish suggests that the involvement of this region in controlling appetite is well conserved. The hypothalamic nuclei expressing *cart2b* mRNA (Figure 5e,h) in Atlantic salmon are consistent with previous studies of hypothalamic *cart* expression (Akash et al., 2014; Porter et al., 2017). Additionally, salmon hypothalamic *cart2b* expression has been shown to respond to a fed/fasted state (Kalananthan et al., 2021). Atlantic salmon hypothalamic neurons also expressed *cart3a* in the NMH area. Indeed, it has been shown that *cart3a* expression is upregulated in the hypothalamus after 3 days of fasting, indicating a potential role in modulating appetite control (Kalananthan et al., 2021). In zebrafish, *cart2a* (previously named *cart2*) presence in the NRL indicated a role in mediating energy homeostasis (Akash et al., 2014). Thus, the expression of *cart2a* in the salmon PTN near the infundibulum supports the observations that *cart2a* might modulate food intake in salmon (Kalananthan et al., 2021). In Atlantic salmon, *cart2* (*cart2a* and *2b*) seems to be the only *cart* gene with similar potential proteolytic sites as its mammalian homolog, based on their sequence alignment (Kalananthan et al., 2021). POMC is a key regulator in the melanocortin system that is post‐transcriptionally cleaved into α‐ and β‐melanocyte‐stimulating hormones, adrenocorticotropic hormone, and β‐endorphin (Takahashi & Mizusawa, 2013). Here, *pomca* was expressed in the NLT area in the brain of Atlantic salmon parr. This result is in line with previous studies of *pomc* or α‐melanocyte‐stimulating hormones in goldfish (Cerdá‐Reverter, Schiöth, et al., 2003; Forlano & Cone, 2007; Porter et al., 2017), barfin flounder *Verasper moseri* (Amano et al., 2005), zebrafish (Zhang et al., 2012), African cichlid fish (Porter et al., 2017), and rainbow trout (Otero‐Rodino et al., 2019). These observations together with the findings of Kalananthan, Murashita, et al. (2020) suggest the involvement of *pomca* in Atlantic salmon appetite control.

### 4.2 Expression of melanocortin system neuropeptides in other brain regions

The widespread distribution of *npy* and *cart* in the brain of Atlantic salmon parr indicates various potential functional roles in the central nervous system. The neuropeptides *npya* and *cart2b* were the most abundant, as previously demonstrated (Kalananthan et al., 2021; Tolås et al., 2021). Additionally, *cart2b* expression resembled that of *npy* in brain areas associated with sensory processing, such as its presence in the olfactory bulb, which is known to be linked with processing chemosensory information, immune responses, and reproduction (Ye et al., 2020). This expression is consistent with other studies in teleosts for *npy* (Cerdá‐Reverter et al., 2000; Gaikwad et al., 2004; Kaniganti et al., 2021; Pirone et al., 2008; Porter et al., 2017) and *cart* or *cart2b* (Akash et al., 2014; Bonacic et al., 2015; Kalananthan et al., 2021; Le et al., 2016). Interestingly, fasted zebrafish express higher levels of *npy* in the olfactory bulb compared to those fed (Kaniganti et al., 2021), while in Atlantic salmon fasting decreased *npya1* and increased *cart2b* mRNA levels in the olfactory bulb (Kalananthan et al., 2021; Tolås et al., 2021). In fact, *npy* has been suggested to serve as a neurotransmitter, while *cart* is involved in modulating the activity that can affect chemosensory processing and food‐seeking behavior (Akash et al., 2014; Gaikwad et al., 2004; Kalananthan et al., 2021; Kaniganti et al., 2021; Singru et al., 2008; Tolås et al., 2021). Taken together, these two peptides (*npya* and *cart2b*) may work together or independently in processing and transmitting olfactory sensory information in Atlantic salmon.

*npy* is expressed in the telencephalon of all teleost species investigated to date (Castro et al., 1999; Cerdá‐Reverter et al., 2000; Gaikwad et al., 2004; Le et al., 2016; Pirone et al., 2008; Porter et al., 2017; Saha et al., 2015; Singru et al., 2008; Tolås et al., 2021). In the ventral telencephalon, *npya* was abundantly expressed (Figure 1c), while *npyb* was much less abundant (Figure 2a). Further, *cart2a*, *2b*, and *3a* were also expressed in the ventral telencephalon (Figures 5, 6, and 8b). Telencephalon plays a role in sensory input processing connected to various functions such as reproduction (Saha et al., 2015; Uezono et al., 2015), behavior (Comesaña et al., 2018), and appetite control (Ye et al., 2020). Anatomically, the telencephalon has afferent and efferent connections with many brain regions, including the olfactory bulb, preoptic region, and tuberal hypothalamus (Folgueira et al., 2004a, 2004b). Telencephalic *cart* expression is linked with sensory‐motor function, while *npy* expression in the telencephalon has been linked to olfactory sensory processing (Singru et al., 2008), suggesting that *npy* might be involved in the hedonic control of food intake in this brain region. Interestingly, zebrafish *cart2* (Akash et al., 2014) and catfish *cart* (Subhedar et al., 2011) decrease in the entopeduncular nucleus during starvation, while fasting had no impact on Atlantic salmon *npy* and *cart* transcripts in the telencephalon (Kalananthan et al., 2021; Tolås et al., 2021). The species‐specific *cart* responses indicate that more research is needed to understand the role of telencephalic *cart* in appetite control in teleost species. *agrp2* was strongly expressed in the dorsal telencephalon in Atlantic salmon parr (Figure 4a), which is in line with previous findings in salmon (Kalananthan, Lai, et al., 2020). Opposite to that found in zebrafish, no *agpr2* expression was observed in the pineal cells of Atlantic salmon pa (Shainer et al., 2017, 2019; Zhang et al., 2010). Zebrafish *agrp2* has been found in novel, uncharacterized, nonphotosensitive pineal cells, in addition to a few neurons in the preoptic region that project to the adenohypophysis, indicating that this neuropeptide is linked to the hypophysiotropic stress axis in zebrafish (Shainer et al., 2017). As suggested in zebrafish, *agrp2* in salmon might have a functional role in the spatial navigation network or a stress response via cortisol and the medial and lateral zones of the dorsal telencephalic serotonergic system (Rodríguez et al., 2021; Silva et al., 2015).

The preoptic region, located rostral to the hypothalamus, is functionally and neurochemically associated with the hypothalamus—including reproduction and sensory processing (Porter et al., 2017). In fact, the preoptic region functions as a key region for downstream signaling as the neurons from the preoptic region may be connected to several brain regions (Folgueira et al., 2004b), and innervate the pituitary via the hypothalamic‐neurohypophyseal tract (Akash et al., 2014; Forlano & Cone, 2007; Herget et al., 2014). These signals include serotonergic and corticotropin‐releasing factor systems that can affect food intake (Ortega et al., 2013). Preoptic expression of Atlantic salmon *npya* was observed in several subregions, including the SOC (Figure 1e), as it has previously been shown for other teleost species (Cerdá‐Reverter et al., 2000; Jeong et al., 2018; Le et al., 2016; Perez Sirkin et al., 2013; Pirone et al., 2008; Porter et al., 2017). Moreover, *cart2b*, *3a*, *3b*, and *4* were expressed in the preoptic region (Figures 4, 5, 6b, and 8a), similar to that reported for other teleosts (Akash et al., 2014; Le et al., 2016; Mukherjee et al., 2012; Porter et al., 2017), suggesting that *cart*, like *npya*, might be involved as preoptic neuroendocrine regulators. *npya* was expressed ventrally in the left and right habenula (Figure 1f), which is homologous to the mammalian lateral habenula (Amo et al., 2010) where NPY modulates excitatory transmissions (Cheon et al., 2019). Moreover, lateral habenular NPY might be indirectly linked to the hedonic regulation of appetite in primates (reviewed by Rezitis et al. (2022)). The lateral habenula is indeed a central node connecting rostral and caudal brain regions; afferent connections originate from the nucleus entopeduncularis (ENT, homologous to the globus pallidus in primates), and efferent connections to the median raphe nucleus in the ventral tegmentum (Hikosaka et al., 2008; Turner et al., 2016). Furthermore, *agrp2*, *cart2a*, *2b*, *3a*, *3b*, and *npya* were expressed in the thalamus (Figures 1, 4, 6e,d, 7c, and 8d), suggesting an involvement of these neuropeptides in the modulation of sensory inputs to the telencephalon (Folgueira et al., 2004a, 2004b; Singru et al., 2007). In the midbrain, *npyb* was expressed exclusively near NPPv as well as in proximity to NIII (Figure 2b), with no expression observed ventrally toward the hypothalamus. This suggests that *npyb* might not have a direct role in appetite control, which agrees with previous studies on Atlantic salmon (Tolås et al., 2021), tiger puffer *Takifugu rubripes* (Kamijo et al., 2011), and Nile tilapia *Oreochromis niloticus* (Yan et al., 2017).

In zebrafish, visual information is essential to modulate feeding behavior (Muto et al., 2017), and feeding state modulates the activity of sensory processing involved in fine‐tuning the response to external stimuli, such as prey capture or avoidance behavior (Corradi & Filosa, 2021). Atlantic salmon *npya* expression in the SPV and scattered cells in SGV of the optic tectum (Figure 1g,l) was in line with previous studies in teleost species (Cerdá‐Reverter et al., 2000; Das et al., 2019; Porter et al., 2017). Together, this indicates that *npya* may have a role in both signaling feeding status and visual perception, as suggested previously for Atlantic salmon (Tolås et al., 2021) and zebrafish (Filosa et al., 2016), and also supported by the high expression of *npy* in the salmon eye (Murashita et al., 2009). In the optic tectum, *cart2b* expressed in the SPV (Figure 5e,a,i) was similar to that of *npya*, while *cart2a* and *3a* were expressed in the distal layers of SPV and torus longitudinalis, respectively, (Figures 6d–f and 8d,e) suggesting a role in integrating visual information for the later (Filosa et al., 2016).

In mammals, the EW plays a vital role in the integration and modulation of sympathetic outflow affecting stress and energy homeostasis through orexigenic and anorexigenic projections from the hypothalamic arcuate nucleus and paraventricular nucleus (Cano et al., 2021). In contrast, the EW in teleosts is rarely connected to appetite but it is described to be photosensitive in zebrafish (Hang et al., 2014). In this study, *npya* and *cart3a* were found near EW (Figures 1, 6), which is in line with previous studies for *cart* in teleosts, including catfish (Singru et al., 2007) and zebrafish (Akash et al., 2014), and this indicates that further studies are needed to better understand this region. Laminated TS receives inputs from the lateral line and visual system (Pirone et al., 2008), suggesting that *cart2b* and *3b* might be involved in the processing of both visual and lateral stimuli. In the rhombencephalon, *npya*, *b*, *agrp2*, *cart1a*, *3a*, and *3b* were observed proximally to the FLM and nV. The *npy* expression observed here is similar to previous findings in Atlantic salmon and *Gambusia affinis* by NPY‐immunoreactivity (Garcia‐Fernandez et al., 1992). The expression of these neuropeptides near the nV indicates a possible involvement in food intake and sensory inputs from the oral cavity (Pirone et al., 2008). The expression of *cart1a*, *3a*, and *3b* in the rhombencephalic region suggests that these neurons may innervate the spinal cord and, thus, these neuropeptides may play a role in descending control from the brain stem, as speculated for *cart* in zebrafish (Akash et al., 2014).

The main site for *pomc* expression was the adenohypophysis, in line with previous observations in Atlantic salmon by qPCR (Kalananthan, Lai, et al., 2020; Kalananthan, Murashita, et al., 2020) and other teleost species (Amano et al., 2005; Forlano & Cone, 2007; Otero‐Rodino et al., 2019; Zhang et al., 2012). Downstream signaling from the adrenocorticotropic hormone, one peptide produced from *pomc*, is the hypothalamus‐pituitary‐interrenal axis affecting food intake through glucocorticoid production. Interestingly, starved zebrafish have been shown to have lower cortisol levels than fed fish (Filosa et al., 2016). Downstream signaling from the melanocyte‐stimulating hormones includes physiological color change mechanisms and stress response (Segura‐Noguera et al., 2000) that indirectly affect food intake. Mapping hypophysiotropic neurons in the hypothalamus by immunocytochemical studies has shown that α‐melanocyte‐stimulating hormone fibers project from NLT down to the pituitary in zebrafish (Zhang et al., 2012), but not in barfin flounder (Amano et al., 2005). The contradictory effects of *pomca* observed in previous studies might be explained by the end‐product of the post‐translational cleavage of *pomc*. While α‐melanocyte‐stimulating hormone has been shown to be a direct suppressor of appetite, β‐endorphin can antagonize the α‐melanocyte‐stimulating hormone downstream signaling pathways directly (Mercer et al., 2013). Thus, more research is needed to investigate the relationship between *pomc* and appetite and energy balance in vertebrates.

## 5 CONCLUSION

This study shows that the Atlantic salmon neuropeptides *npy*, *cart*, *pomc*, and *npy* are expressed in brain regions known to be related to feeding and energy status. This includes the hypothalamus, supporting the hypothesis that the melanocortin system and the NLT region of the hypothalamus are involved in the control of appetite in Atlantic salmon and that this function is conserved across vertebrates. In the Atlantic salmon hypothalamus, a distinct neuronal *npya*, *agrp1*, *cart2b*, and *pomca* expression was found, as well as a few neurons coexpressing *cart2b/pomca*. To what extent does this hypothalamic coexpression affect the physiological regulation of food intake compared to the distinct expression of these neuropeptides in Atlantic salmon is a question that needs further investigation. In addition, our data suggest that several of the neuropeptides investigated might be involved in the control of food intake and energy homeostasis through transmission and processing of sensory signals. This is based on their mRNA expression profile in the olfactory bulb, telencephalon, midbrain, and hindbrain.

## AUTHOR CONTRIBUTIONS

*Conceptualization*: Ivar Rønnestad and Jon Vidar Helvik. *Sampling*: Sissel Norland, Mariann Eilertsen, Jon Vidar Helvik and Ana S. Gomes. *Methodology*, *investigation*, *and analysis*: Sissel Norland, Mariann Eilertsen, Jon Vidar Helvik, and Ana S. Gomes. *Writing‐original draft and review and editing*: Sissel Norland, Ivar Rønnestad, Mariann Eilertsen, Jon Vidar Helvik, and Ana S. Gomes.

## CONFLICT OF INTEREST

The authors declare no conflict of interest.

## Data Availability

The data supporting the findings of this paper are primarily presented within the scope of this publication. Additional materials are available upon reasonable request to the corresponding author.

## ENDNOTE

1
https://www.ncbi.nlm.nih.gov/genbank/.
